# Microbial Legacy: *Mycobacterium vaccae* ATCC 15483^T^ intergenerationally diversifies the microbiome and enhances stress resilience in male mice

**DOI:** 10.1038/s41380-026-03638-9

**Published:** 2026-05-22

**Authors:** Jessica Schiele, Pei-Ling Tsai, Tamara Schimmele, Sina Beck, Maren Meyer, Marco Mannes, Luke W. Desmond, Reiner Noschka, Markus Huber-Lang, Melanie Haffner-Luntzer, Marc N. Jarczok, Christopher A. Lowry, Andreas Reif, Dominik Langgartner, Steffen Stenger, David A. Slattery, Stefan O. Reber

**Affiliations:** 1 Laboratory for Molecular Psychosomatics, Department of Psychosomatic Medicine and Psychotherapy, Ulm University Medical Center, 89081 Ulm, Germany; 2 Goethe University Frankfurt, University Hospital, Department of Psychiatry, Psychosomatic Medicine and Psychotherapy, 60528 Frankfurt, Germany; 3 Institute of Orthopaedic Research and Biomechanics, Ulm University Medical Center, 89081 Ulm, Germany; 4 Institute of Clinical and Experimental Trauma-Immunology, Ulm University Medical Center, 89081 Ulm, Germany; 5 Department of Integrative Physiology, Department of Psychology and Neuroscience, Center for Neuroscience and Center for Microbial Exploration, University of Colorado Boulder, Boulder, CO 80309 USA; 6 Institute of Medical Microbiology and Hygiene, Ulm University Medical Center, 89081 Ulm, Germany; 7 Department of Psychosomatic Medicine and Psychotherapy, Ulm University Medical Center, 89081 Ulm, Germany; 8 German Center for Mental Health (DZPG), partner site Mannheim//-Heidelberg//-Ulm, Ulm, Germany

**Keywords:** Molecular biology, Psychiatric disorders

## Abstract

According to the “Old Friends” hypothesis, the increased prevalence of stress-associated disorders in urban concrete landscapes of high-income countries is at least in part due to a reduced exposure to immunoregulatory microorganisms. The latter is particularly impactful when occurring during early prenatal and postnatal life. Accordingly, our own preclinical studies demonstrate that non-pathogenic rapid-growing mycobacteria, including *Mycobacterium (M.) vaccae* NCTC 11659 and *M. vaccae* ATCC 15483^T^, have immunoregulatory and stress-protective effects when administered repeatedly prior to or during stressor exposure. Here, we advance these findings by showing that repeated intragastric (i.g.) administration of a heat-killed preparation of *M. vaccae* ATCC 15483^T^ to female C57BL/6 N mice provides intergenerational stress protection. Their male offspring, despite never directly receiving administration of rapid-growing mycobacteria, were protected against multiple adverse consequences of chronic stress in adulthood. Moreover, correlational analyses implicate the fecal microbiome as a potential mediator of these effects, with *M. vaccae* ATCC 15483^T^ intergenerationally facilitating α-diversity and increasing the relative abundance of bacterial taxa known to be potent short-chain fatty acid producers.

**Figure:**
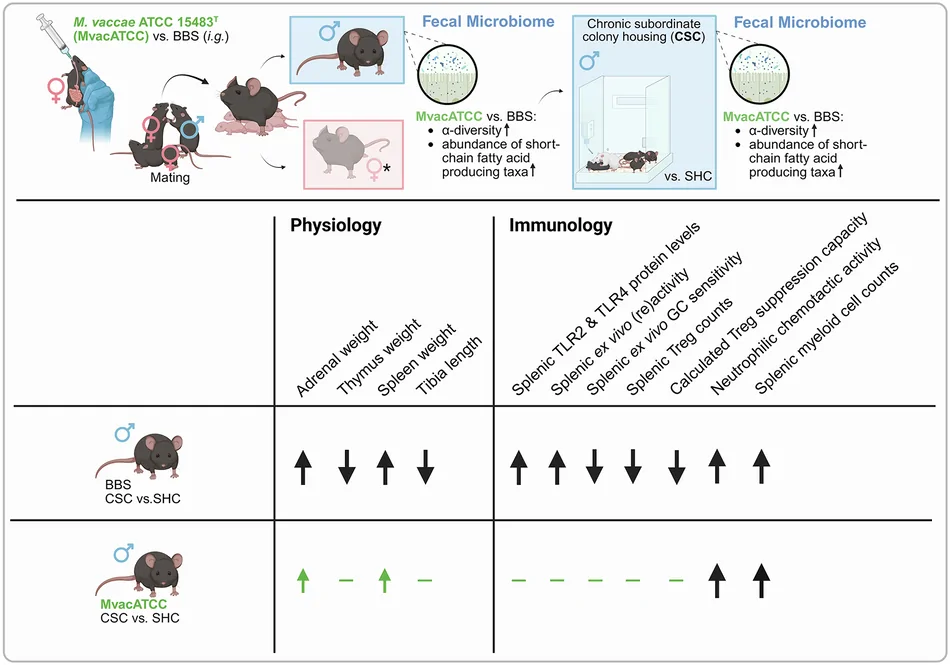
Repeated intragastric (i.g.) administration of a heat-killed preparation of *Mycobacterium (M.) vaccae* ATCC 15483^T^ (MvacATCC)vs. its vehicle borate-buffered saline (BBS) to adult nulliparous female C57BL/6N mice was intergenerationally protective against multiple negative physiological and immunological consequences of chronic subordinate colony housing (CSC; compared with respective single-housed control (SHC) mice), including adrenal hypertrophy, splenomegaly, thymus involution, and tibia growth reduction as well as increased splenic toll-like receptor (TLR) 2 and TLR4 protein concentrations and splenocyte ex vivo (re)activity, but also decreased splenic ex vivo glucocorticoid  sensitivity, regulatory T cell (Treg) counts and Treg suppression capacity in their male offspring. In contrast, CSC-induced increase in splenic myeloid cell counts as well as of neutrophilic chemotactic activity was not affected intergenerationally by MvacATCC. Moreover, fecal microbiome analyses before and after CSC showed that MvacATCC intergenerationally facilitated α-diversity and relative abundance of bacterial taxa known to be potent short-chain fatty acid (SCFA) producers. Of note, we abstained from showing respective data of female offspring in the graphical abstract (*), as the intergenerational resilience effects of MvacATCC on female offspring were difficult to interpret. The latter was due to the fact that chronic adult stressor exposure (i.e., social instability paradigm, SIP) per se did not affect any of the physiological and immunological readouts reported in females. The graphical abstract was created with Biorender.com.

Repeated intragastric (i.g.) administration of a heat-killed preparation of *Mycobacterium (M.) vaccae* ATCC 15483^T^ (MvacATCC)vs. its vehicle borate-buffered saline (BBS) to adult nulliparous female C57BL/6N mice was intergenerationally protective against multiple negative physiological and immunological consequences of chronic subordinate colony housing (CSC; compared with respective single-housed control (SHC) mice), including adrenal hypertrophy, splenomegaly, thymus involution, and tibia growth reduction as well as increased splenic toll-like receptor (TLR) 2 and TLR4 protein concentrations and splenocyte ex vivo (re)activity, but also decreased splenic ex vivo glucocorticoid  sensitivity, regulatory T cell (Treg) counts and Treg suppression capacity in their male offspring. In contrast, CSC-induced increase in splenic myeloid cell counts as well as of neutrophilic chemotactic activity was not affected intergenerationally by MvacATCC. Moreover, fecal microbiome analyses before and after CSC showed that MvacATCC intergenerationally facilitated α-diversity and relative abundance of bacterial taxa known to be potent short-chain fatty acid (SCFA) producers. Of note, we abstained from showing respective data of female offspring in the graphical abstract (*), as the intergenerational resilience effects of MvacATCC on female offspring were difficult to interpret. The latter was due to the fact that chronic adult stressor exposure (i.e., social instability paradigm, SIP) per se did not affect any of the physiological and immunological readouts reported in females. The graphical abstract was created with Biorender.com.

## Introduction

Urbanization is on the rise [1], and various stress-associated somatic and mental disorders are more prevalent in urban versus (vs.) rural areas of the developed world [2–4]. Many of these disorders are accompanied by chronic low-grade inflammation [5, 6]. Prospective human and mechanistic animal studies even strengthen the idea that an exaggerated immune (re)activity plays a causal role in their pathogenesis [5, 7–9]. According to the “Old Friends” hypothesis, a reduced exposure to microorganisms with which mammals co-evolved contributes to deficits in immunoregulation. The latter is particularly evident in urban concrete landscapes of high-income countries and when reduced microbial contact occurs during early life [10–13]. Of particular importance are findings highlighting that not just early postnatal [3] but also prenatal (i.e., of the pregnant dam) [14, 15] exposure to “Old Friends” intergenerationally protects the infant against stress-associated disorders. For example, a growing body of evidence indicates that maternal probiotic and prebiotic supplementation during gestation has beneficial effects on the offspring’s stress vulnerability, behavior and immunity [16–20]. Not known in this context is, at least to our knowledge, whether “Old Friends” exposure of the male and/or female F0 generation prior to mating and pregnancy intergenerationally boosts stress resilience in the F1 generation. According to Klengel and colleagues, an intergenerational transfer of effects implies direct exposure of either the unborn F1 generation (if the exposure occurs after conception) or adult germ cells (if the exposure occurs before conception) [21].

Consistent with what is proposed by the “Old Friends” hypothesis, we and others have previously shown that repeated subcutaneous immunizations with a heat-killed preparation of *Mycobacterium (M.) vaccae* NCTC (National Collection of Type Cultures) 11659 facilitates stress resilience in the same generation [22–26]. *M. vaccae* NCTC 11659 is a rapidly growing, aerobic, Gram-positive, acid-alcohol-fast, rod-shaped soil saprophyte, which has been isolated from mud. It forms rough yellow pigmented colonies in culture and has anti-inflammatory and immunoregulatory properties [27, 28]. In terms of the mechanisms underlying its immunoregulatory effects, depletion of CD25^+^ immune cells after the last subcutaneous *M. vaccae* NCTC 11659 administration and prior to stressor exposure revealed that *M. vaccae* NCTC 11659 mediates its stress-protective effects, at least partly *via* induction of Tregs and interleukin (IL)-10 secretion [25]. However, the effects of *M. vaccae* NCTC 11659 on Treg functionality have not been investigated to date [27]. Importantly, our own recent preclinical findings confirm the stress-protective effects of *M. vaccae* NCTC 11659 when administered *via* the non-invasive intranasal or intragastric (i.g.) route [29, 30]. Moreover, we have shown that not just *M. vaccae* NCTC 11659 but also the closely related strain *M. vaccae* ATCC (American Type Culture Collection) 15483^T^ has immunoregulatory and stress-protective effects when administered repeatedly *via* the subcutaneous or i.g. route [24, 31, 32].

The current study aimed to test the hypotheses that *M. vaccae* ATCC 15483^T^, administered to female mice prior to mating, in an intergenerational manner, enhances resilience to chronic psychosocial stress in their male and female offspring, and that this is associated with intergenerational changes in the composition of the fecal microbiome. To quantify possible intergenerational stress resilience effects of *M. vaccae* ATCC 15483^T^, we assessed parameters that have been shown earlier to be negatively affected by the chronic stress paradigms we use in the current study (i.e., increased general and social anxiety-related behavior, adrenal hypertrophy, thymus atrophy, splenomegaly, reduced tibia length, splenic myelocytosis, splenic toll-like receptor (TLR) upregulation, splenocyte activation and glucocorticoid (GC) resistance ex vivo, neutrophil chemotactic hyperactivity, decline in splenic Tregs). Given that, in the present study, oocytes of female mice that received heat-inactivated *M. vaccae* ATCC 15483^T^ before mating were directly exposed to the compound, we refer to its stress-protective effects on the F1 generation as intergenerational effects [21].

## Materials and methods

### Animals

Nulliparous C57BL/6N female (9 weeks, 15 – 20 g) and male mice (9 weeks, 21 – 27 g; used for two mating rounds) purchased from Charles River (Sulzfeld, Germany) were used to obtain in-house bred experimental C57BL/6N male and female mice. CD-1 male mice (30 – 35 g; Charles River) were utilized as dominant aggressors in the chronic subordinate colony housing (CSC) paradigm. All mice were kept under standard specific-pathogen-free laboratory housing conditions (12 h light-dark cycle; lights on at 0600 a.m. (wintertime) or 0700 a.m. (summertime); 22 °C and 60 % humidity) in standardized polycarbonate mouse cages (22 cm length × 16 cm width × 14 cm height) with free access to tap water and standard mouse diet if not stated differently below. All experimental protocols were approved by the Committee on Animal Health and Care of the local government (Regierungspräsidium Tübingen, Germany; TVA-No. 1594) and performed in accordance with national and international guidelines on the ethical use of animals (ARRIVE guidelines 2.0 and EU Directive 2010/63/EU for animal experiments) [33]. Sample size was determined using G*Power 3.1.9.2 (Freeware provided by Duesseldorf University) based on previous publications assessing the stress-protective effects of rapid-growing mycobacteria [30]. All efforts were made to minimize the number of animals used and their suffering.

### Experimental procedures

**Fig. 1:**
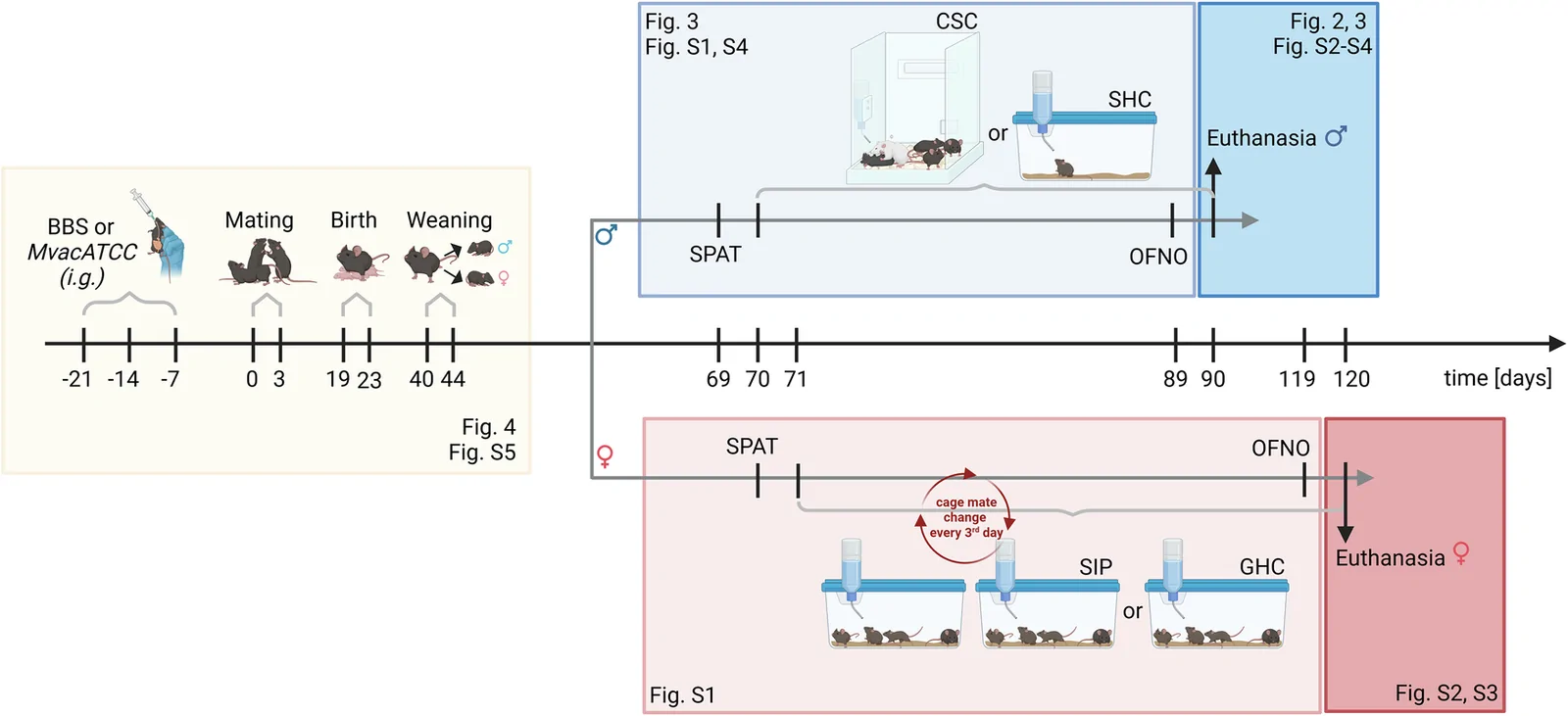
Experimental Timeline – Experiment 1. Adult, nulliparous female C57BL/6N mice received three weekly intragastric (i.g.) administrations of *M. vaccae* ATCC 15483^T^ (MvacATCC) or borate-buffered saline (BBS) prior to mating. After giving birth and weaning, the male (blue boxes) and female (red boxes) offspring were subsequently exposed to either chronic psychosocial stress (males: chronic subordinate colony housing, CSC; females: social instability paradigm, SIP) or respective control conditions (males: single-housed controls, SHC; females: group-housed controls, GHC) with behavioral testing prior (i.e., social preference/avoidance test, SPAT) and after (i.e., open-field/novel object test, OF/NO) chronic psychosocial stressor exposure (for details see Materials and Methods section). Data referring to female C57BL/6N dams can be found in Fig. 4, Fig. S5 and Tabs. S1, S2. Data referring to offspring can be found in Figs. 2, 3, Figs. S1-S4, and Tabs. S1, S2. The experimental timeline was created with Biorender.com.

For the generation of offspring in **Experiment 1** (Fig. 1), nulliparous female C57BL/6N mice were housed in groups of four upon arrival at experimental day –21 (the first day of mating was defined as experimental day 0). All females received three i.g. administrations of vehicle (i.e., borate-buffered saline, BBS) or a heat-killed preparation of *M. vaccae* ATCC 15483^T^ (once per week) before being mated with breeding males between experimental days 0–3. Before giving birth on experimental days 19–23, all pregnant females were housed singly (number of dams/litters per group, Tab. S1; Fig. S5). Maternal “on nest” behavior was observed daily for one week following birth, except on postnatal day 3, when the pup retrieval test was performed (Fig. S5). All litters were housed together with their dam until weaning and sex determination on postnatal day 21, i.e., experimental days 40 and 44 (Fig. 1, yellow box; Fig. S5). After weaning, all dams were tested for general and social anxiety-related behavior as well as for anhedonia-like behavior before being euthanized by rapid decapitation after brief CO_2_ inhalation (data not shown in the current manuscript). Male and female offspring were group-housed at a maximum of five siblings per cage (separated by sex and treatment, respectively). The following parameters of a subset of dams and their respective litters have been reported earlier by our group: 1) number of pups, and body weight per litter at birth; 2) observation intervals spent on nest; 3) latency to retrieve first pup and latency to place pups back in nest; 4) body weight per litter; and 5) number of male and female pups at weaning [32]. Three weeks after weaning, male offspring were housed singly to avoid hierarchy establishment, while female offspring stayed group-housed at a maximum of four animals per cage. The male and female offspring were randomly assigned to a no-stress control group (males: single-housed controls, SHC; females: group-housed controls, GHC) or a chronic stress group (males: chronic subordinate colony housing, CSC; females: social instability paradigm, SIP) to investigate the intergenerational effects of i.g. *M. vaccae* ATCC 15483^T^ on the consequences of chronic adult stress (Fig. 1, blue and red boxes). Litter effects were minimized by assuring that no more than *N* = 3 pups per sex and litter were assigned to one experimental group (males: BBS-SHC: 9 litters; BBS-CSC: 6 litters; *M. vaccae* ATCC 15483^T^-SHC: 6 litters; *M. vaccae* ATCC 15483^T^-CSC: 5 litters; females: BBS-GHC: 10 litters; BBS-SIP: 6 litters; *M. vaccae* ATCC 15483^T^-GHC: 5 litters; *M. vaccae* ATCC 15483^T^-SIP: 5 litters).

**Fig. 2:**
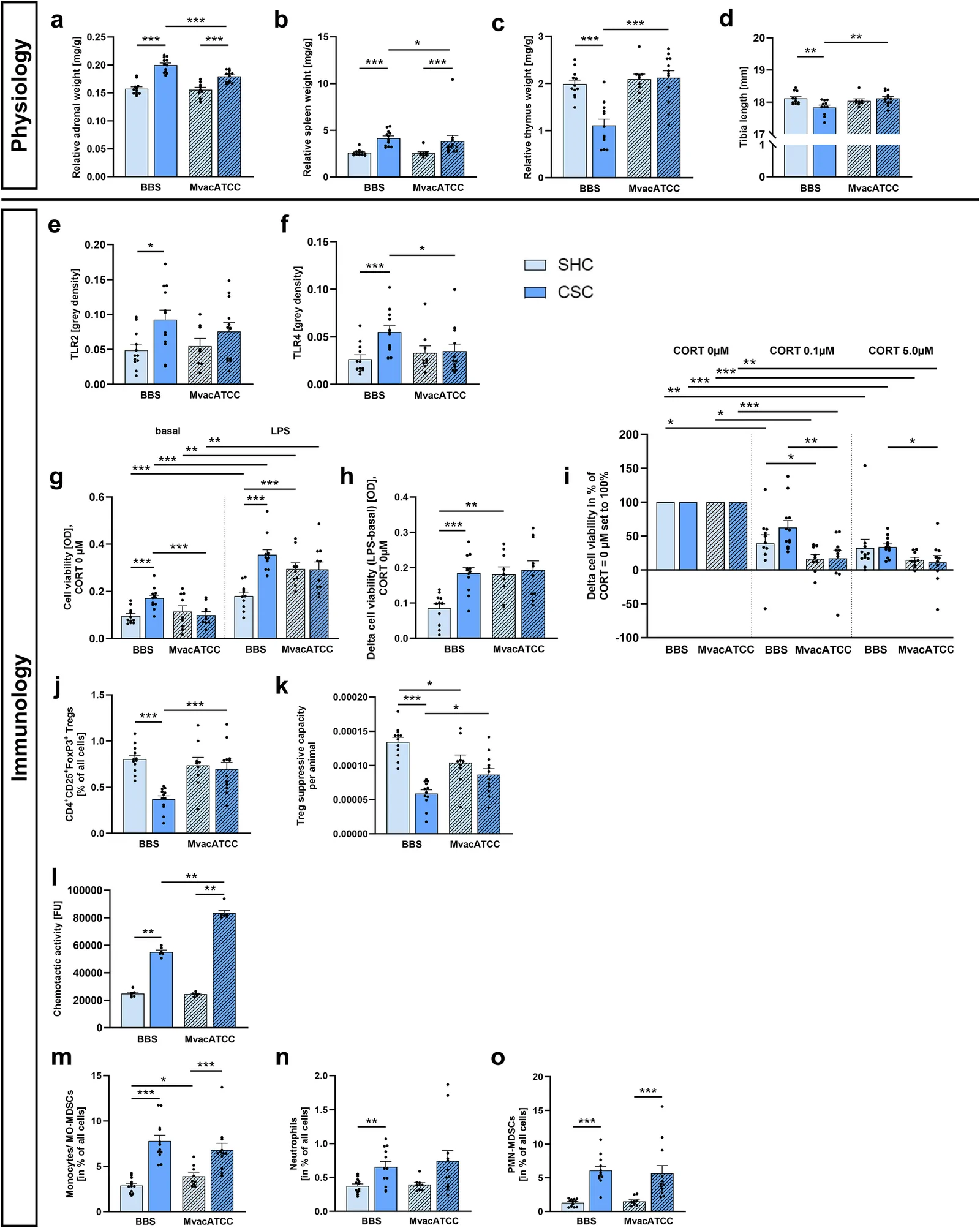
Intergenerational effects of repeated i.g. administration of *Mycobacterium vaccae* ATCC 15483^T^ to adult, nulliparous female C57BL/6N mice prior to mating on the negative consequences of chronic psychosocial stress during adulthood in male offspring (Experiment 1). **a-o** show physiological and immunological data from experimental male offspring exposed to either chronic psychosocial stress (chronic subordinate colony housing, CSC) or no-stress condition (single-housed controls, SHCs) during adulthood. Relative (**a**) adrenal, (**b**) spleen, and (**c**) thymus weight. **d** Tibia length. **e** Splenic toll-like receptor (TLR)2, and (**f**) TLR4 protein levels. **g** Ex vivo cell viability of isolated splenocytes under basal and lipopolysaccharide (LPS) conditions at 0 µM corticosterone (CORT). **h** Delta (LPS minus basal) ex vivo splenocyte viability at 0 µM CORT. **i** Delta ex vivo splenocyte viability at 0.1 µM and 5 µM CORT in percent (%) of 0 µM CORT set to 100%. **j** Splenic CD4^+^CD25^+^FoxP3^+^ regulatory T cells (Tregs) in percent (%) of all splenocytes, (**k**) calculated Treg suppressive capacity per male experimental mouse. **l** Chemotactic activity of isolated neutrophils (*N* = 6 technical replicates per group). Percentage of splenic (**m**) CD11b^+^Ly6G^–^Ly6C^+^ monocytes/monocyte-like (MO) myeloid-derived suppressor cells (MDSCs), (**n**) CD11b^+^Ly6G^+^Ly6C^–^ neutrophils, (**o**) CD11b^+^Ly6G^+^Ly6C^+^ polymorphonuclear (PMN)-MDSCs. Data are presented as mean + SEM including individual values (dots). Non-hatched bars: borate-buffered saline (BBS), hatched bars: *M. vaccae* ATCC 15483^T^ (MvacATCC). * *P* ≤ 0.05, ** *P* ≤ 0.01, *** *P* ≤ 0.001 vs. respective control condition. *N* = 8 – 12 per group (Tab. S1). In case individual samples had to be excluded due to methodological reasons, this is reported in the materials and methods. Exact *P* values and statistical tests employed are provided in Tab. S2.

**Fig. 3:**
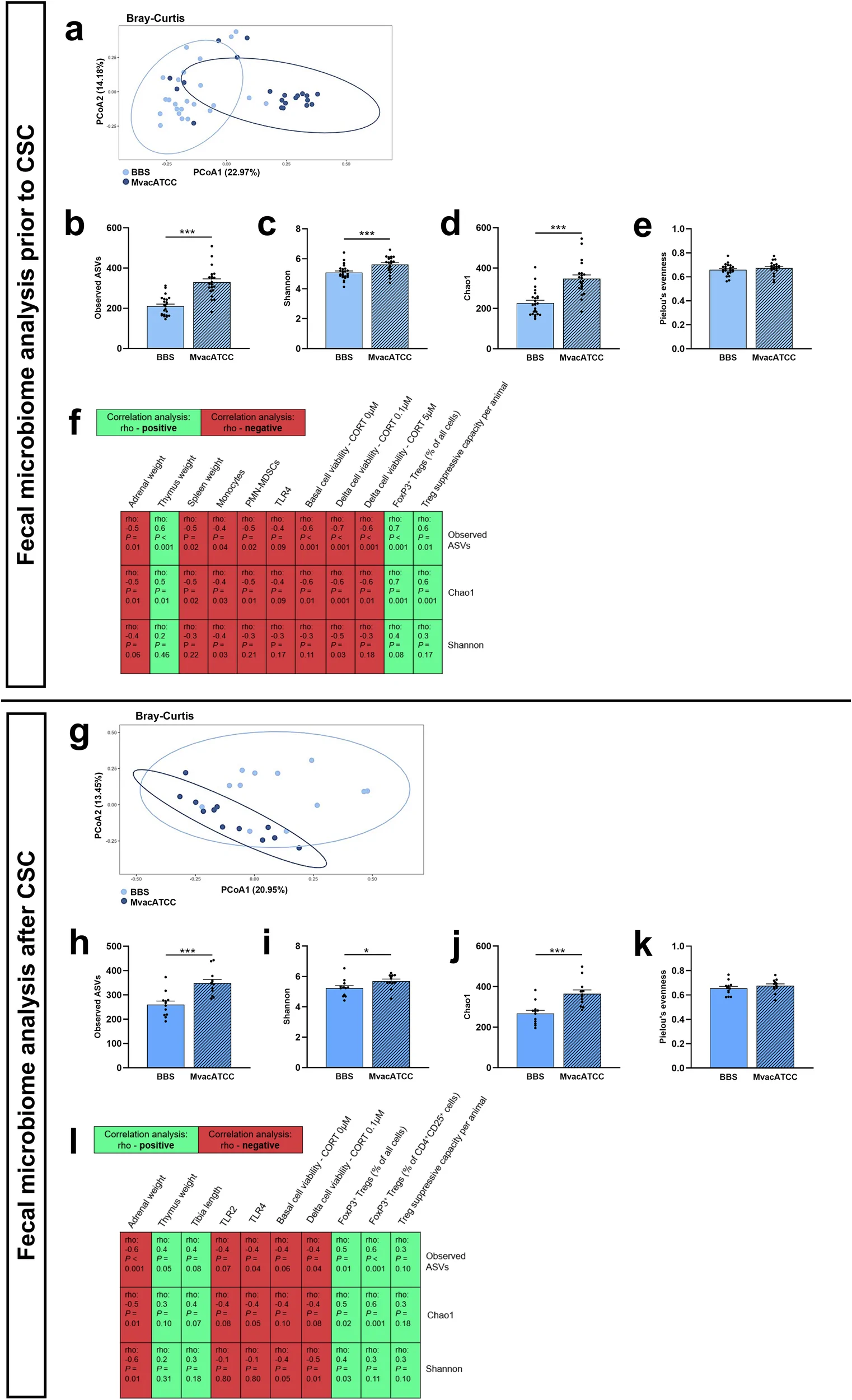
Intergenerational effects of repeated i.g. administration of *Mycobacterium vaccae* ATCC 15483^T^ to adult, nulliparous female C57BL/6N mice prior to mating on the diversity of the fecal microbiome in male offspring prior to and after the exposure to chronic psychosocial stress in adulthood (Experiment 1). Fecal microbiome analyses in male offspring prior to (**a-f**) and after (**g-l**) chronic psychosocial stress exposure. **a,** **g** β-diversity (Bray-Curtis) principal coordinates analysis (PCoA). **b-e,** **h-k** α-diversity metrics (**b,** **h**) observed amplicon sequence variants (ASVs), (**c,** **i**) Shannon diversity index, (**d,** **j**) Chao1 richness estimate (Chao1) and (**e,** **k**) Pielou’s evenness. **f,** **l** Correlation analysis of α-diversity metrics with physiological and immunological parameters. (abbreviations: polymorphonuclear myeloid-derived suppressor cells (PMN-MDSCs), toll-like receptor (TLR), corticosterone (CORT), regulatory T cells (Tregs)). Data are presented as mean + SEM including individual values (dots). Non-hatched bars: borate-buffered saline (BBS), hatched bars: *M. vaccae* ATCC 15483^T^ (MvacATCC). * *P* ≤ 0.05, *** *P* ≤ 0.001 vs. respective control condition. *N* = 8 – 24 animals per group (Tab. S1). Exact *P* values and statistical tests employed are provided in Tab. S2.

**Fig. 4:**
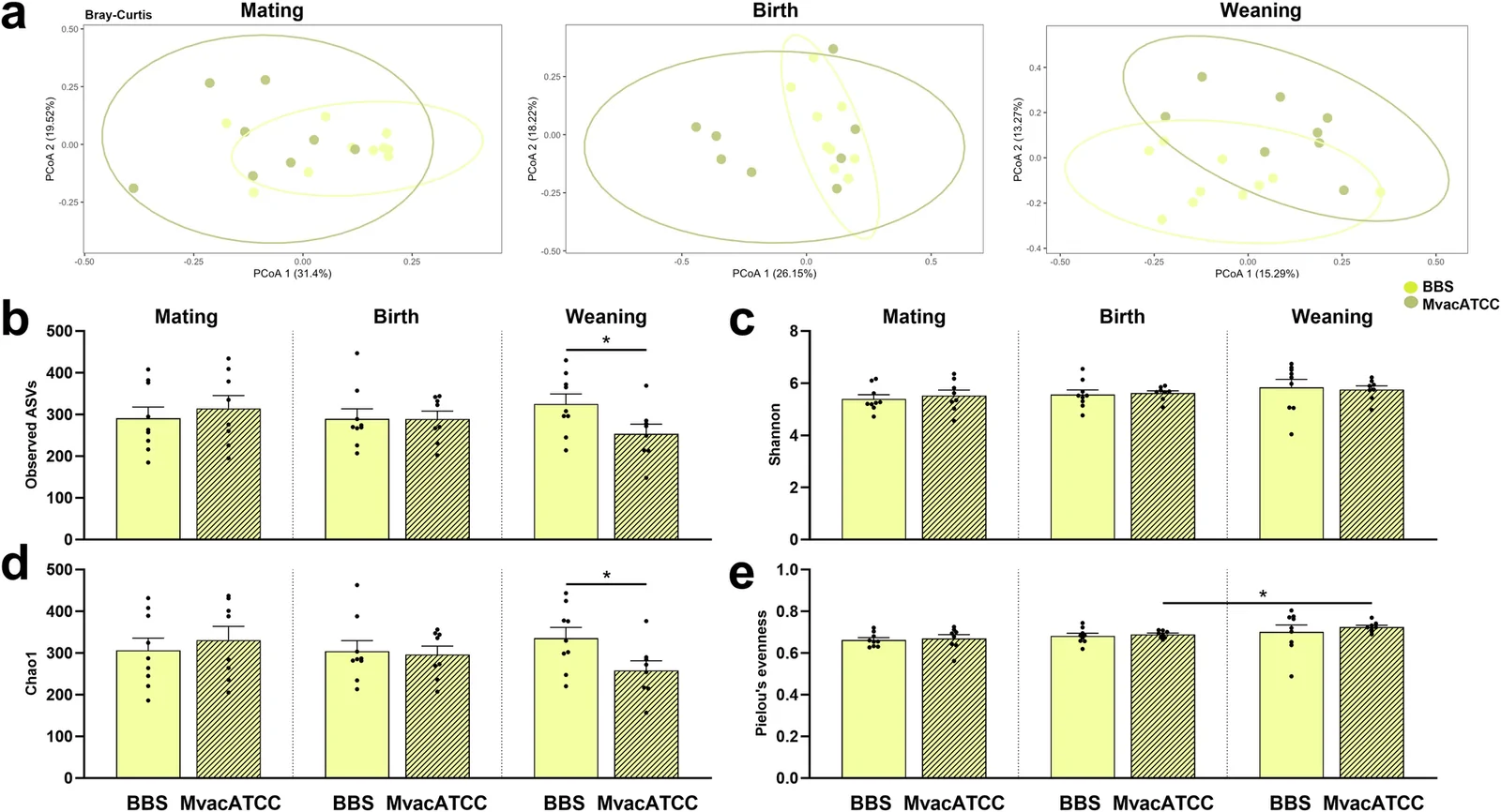
Effects of repeated i.g. administration of *Mycobacterium vaccae* ATCC 15483^T^ to adult, nulliparous female C57BL/6N mice prior to mating on the diversity of their fecal microbiome at mating, birth and weaning (Experiment 1). **a** β-diversity (Bray-Curtis) principal coordinates analysis (PCoA). **b-e** α-diversity metrics, with (**b**) observed amplicon sequence variants (ASVs), (**c**) Shannon diversity index, (**d**) Chao1 richness estimate (Chao1) and (**e**) Pielou’s evenness. Data are presented as mean + SEM including individual values (dots). Non-hatched bars: borate-buffered saline (BBS), hatched bars: *M. vaccae* ATCC 15483^T^ (MvacATCC). * *P* ≤ 0.05 vs. respective control condition. *N* = 10 animals per group. Exact *P* values and statistical tests employed are provided in Tab. S2.

Offspring (number of males and females per group, Tab. S1) were tested for general and social anxiety-related behavior in the social preference/avoidance test one day prior to the start of the chronic adult stress paradigms (Fig. 1, light blue or light red boxes; Fig. S1). At the end of the chronic adult stress paradigms, all male and female mice were tested for general anxiety-related behavior in the open-field/novel object test (Fig. 1, light blue or light red boxes; Fig. S1). One day later, all male and female mice were euthanized by rapid decapitation after brief CO_2_ inhalation for the assessment of physiological, bone-related and immunological parameters that have been earlier shown by our group to be negatively affected by CSC (Fig. 2) and/or SIP [30, 34, 35]. Fecal pellets for analysis of the microbiome composition were collected from total male offspring prior to CSC exposure (Fig. 3; Fig. S4) and from the CSC male offspring after CSC exposure (Fig. 3; Fig. S4) as well as from their respective BBS and *M. vaccae* ATCC 15483^T^ dams at mating, birth and weaning (Figs. 1, 4; Fig. S5h). Importantly, for the following parameters, data from the SHC males of BBS-administered dams have been reported earlier by our group: 1) delta ex vivo cell viability of isolated splenocytes; 2) neutrophil chemotactic activity; 3) social preference; 4) distance moved and entries into inner zone during open field exploration as well as entries into contact zone during novel object exploration; 5) relative adrenal and thymus weights [32].

Finally, in **Experiment 2** nulliparous female mice (Fig. S6a) were housed in groups of four upon arrival at experimental day –21, received three i.g. administrations of BBS or *M. vaccae* ATCC 15483^T^ before being mated with breeding males between experimental days 0–3. On the following day, all females received an intraperitoneal injection with bromodeoxyuridine (50 mg/kg in 0.9% NaCl), as these dams served as pregnancy stress controls for another study assessing pregnancy stress effects on dam’s hippocampal neurogenesis (data not shown in the current manuscript). One to three days after giving birth, dams and their offspring were euthanized and maternal mammary gland tissue was collected for assessment of C3 mRNA expression (number of dams per group, Tab. S1; Fig. S6b).

### Intragastric (i.g.) administration of *M. vaccae* ATCC 15483^T^ or its vehicle borate-buffered saline (BBS)

Heat-inactivated *M. vaccae* ATCC 15483^T^ was provided by the Department of Medical Microbiology and Hygiene of Ulm University Medical Center (Ulm, Germany) as described previously [32]. Briefly, *M. vaccae* ATCC 15483^T^ was cultured at 37 °C and 5 % CO_2_, until confluent growth was visible. Subsequently, bacteria were resuspended in phosphate-buffered saline and heat-inactivated (30 min, 86 °C). After centrifugation, the bacterial pellet was resuspended in BBS and frozen at –80 °C until verification of successful heat-inactivation. Subsequently, the solution was thawed and adjusted to a concentration of 10 mg/mL. Heat-killed bacterial stock was stored at 4 °C until usage. Administrations (i.g.) were performed as described previously [32]. For details, see supplementary materials and methods (Section 1.1).

### Mating procedure, birth and weaning

In order to generate male and female experimental offspring, two C57BL/6N females of each experimental group were mated with one breeding male for four days (experimental days 0–3). Following the end of mating at experimental day 4, females were placed back into their original housing constellation until being housed individually on experimental day 17 to provide a calm and less stressful environment for the upcoming birth of the offspring (experimental days 19–23; Fig. 1).

If a litter was found before 0200 p.m., the same day was assigned as postnatal day 0, if a litter was found after 0200 p.m., the following day was assigned as postnatal day 0. At postnatal day 1, the number of pups and total weight of the entire litter were determined (Tab. S1; Fig. S5). Of note, two *M. vaccae* ATCC 15483^T^-administered dams lost their entire litter before the day of weaning and were therefore not included in the maternal parameters “body weight per litter at weaning” and “number of male and female pups at weaning”.

### Maternal behavior

Maternal behavior was observed daily throughout the first week following birth (postnatal days 1–6, except on postnatal day 3) as described previously with slight modifications [36]. For details, see supplementary materials and methods (Section 1.2).

### Pup retrieval test in dams

All dams were tested for maternal responsiveness on postnatal day 3 using the pup retrieval test based on previously published test procedures [37, 38]. For details, see supplementary materials and methods (Section 1.3).

### Open-field/novel object test

To test for general anxiety-related behavior, the open-field/novel object test [39, 40] was performed as previously published [30]. For details, see supplementary materials and methods (Section 1.4).

### Social preference/avoidance test

General and social anxiety-related behavior was assessed employing the social preference/avoidance test as previously described [30, 41] with minor modifications. For details, see supplementary materials and methods (Section 1.5).

### Chronic subordinate colony housing (CSC) paradigm

The CSC paradigm was conducted as previously described [30]. For details, see supplementary materials and methods (Section 1.6).

### Social instability paradigm (SIP)

To induce chronic psychosocial stress in C57BL/6N female mice, the SIP was conducted as published previously [41]. For details, see supplementary materials and methods (Section 1.7).

### Blood collection

Trunk blood was collected in EDTA-coated tubes (Sarstaedt) following short CO_2_ inhalation and decapitation as previously published [29, 42].

### Organ collection and determination of stress-sensitive organ weights

Following euthanasia, adrenal glands, thymus and spleen were removed, pruned of fat and weighed individually. Spleens were stored in ice-cold Hanks´ balanced salt solution (Sigma-Aldrich, St- Louis, MO, USA) before being used for ex vivo isolation of spleen cells [43].

### Tibia length measurement

Length measurement of the right tibia was performed as previously described [44]. Bones were dissected and stored in 4 % formalin. Tibia length was assessed using a digital precision caliper. Of note, one male offspring of each group in Experiment 1, except for the CSC offspring from *M. vaccae* ATCC 15483^T^-administered dams, was excluded due to technical issues during bone preparation.

### Bite wound severity assessment

To assess the severity of bite wounds in CSC mice, a bite score established by our group was used as previously described [43]. For details, see supplementary materials and methods (Section 1.8).

### Assessment of chemotactic activity of neutrophil/polymorphonuclear (PMN)–myeloid-derived suppressor cell (MDSC)–enriched white blood cells

The chemotactic assay of neutrophil/PMN-MDSC-enriched white blood cells was performed as described previously [32]. For details, see supplementary materials and methods (Section 1.9).

### Ex vivo splenocyte isolation

Splenocytes were isolated as previously described [30]. For details, see supplementary materials and methods (Section 1.10).

### Preparation of cell-protected frozen splenocytes for flow cytometry

Frozen splenocytes were prepared for flow cytometry according to a protocol described in the supplementary materials and methods (Section 1.11).

### Flow cytometry analysis of splenic myeloid subpopulations

Splenocytes were analyzed for the percentages of CD11b^+^Ly6C^+^Ly6G^–^ monocytes (MO)/MO–myeloid derived suppressor cells (MDSCs), CD11b^+^Ly6C^–^Ly6G^+^ neutrophils and CD11b^+^Ly6C^+^Ly6G^+^ polymorphonuclear (PMN)–MDSCs as previously published [30]. For details, see supplementary materials and methods (Section 1.12).

### Flow cytometry analysis of splenic regulatory T cells (Tregs)

Splenocytes were analyzed for the percentages of CD4^+^CD25^+^FoxP3^+^ Tregs as previously described [30]. For details, see supplementary materials and methods (Section 1.13).

### Protein isolation and western blot

To assess splenic expression level of toll-like receptor (TLR)2 and TLR4, protein isolation was performed from ex vivo isolated splenocytes followed by western blot analysis as described previously [30] with minor modifications. For details, see supplementary materials and methods (Section 1.14).

### Functional ex vivo glucocorticoid sensitivity assay of isolated splenocytes

Freshly isolated splenocytes were stimulated with lipopolysaccharide (LPS) and corticosterone to assess functional ex vivo glucocorticoid sensitivity as previously described [30]. For details, see supplementary materials and methods (Section 1.15).

### Treg suppression assay

To investigate suppressive capacity of Tregs, CD4^+^CD25^+^ T cells were isolated and incubated for 48 h with carboxyfluorescein succinimidyl ester-stained responder cells in the presence or absence of anti-CD3/28 before cell proliferation was measured *via* flow cytometry. For details, see supplementary materials and methods (Section 1.16; Fig. S2).

### Preparation for Ultra-High-Throughput Microbial Community Analysis

Microbial community analysis was performed in fecal samples from male offspring of Experiment 1 (collected before SHC/CSC and after the CSC exposure), as well as from BBS- and *M. vaccae* ATCC 15483^T^-administered dams (collected at time points “mating”, “birth” and “weaning”). Noteworthy, Fig. S4a-d and Fig. S5h reporting/correlating relative abundances of bacterial taxa were restricted to taxa that were above the established cut-off for microbial abundance analyses (0.1 %) in the majority of samples [45]. For further details, see supplementary materials and methods (Section 1.17).

### Mammary gland tissue homogenization and assessment of C3 mRNA expression

Mammary gland tissue was freed from hair using a scalpel before 10 mg of tissue were added to 350 µl RLT buffer containing 1 % β-mercaptoethanol in lysis tubes (innuSPEED Lysis Tube P, Innuscreen GmbH). Tubes were placed in a homogenizer (SpeedMill) and samples were homogenized using the program “soft tissue 2”. RNA isolation and RT-qPCR were conducted as previously described [46]. For details, see supplementary materials and methods (Section 1.18).

### Statistical analysis

For statistical analysis and graphical illustrations of data not referred to in the section about microbiome analysis, GraphPad Prism (version 10.1.1, GraphPad Software, LCC), was used as described previously [30]. For details, see supplementary materials and methods (Section 1.19) and Tab. S2.

## Results

To assess the intergenerational stress resilience effects of rapid-growing mycobacteria, BBS as vehicle or a heat-killed preparation of *M. vaccae* ATCC 15483^T^ were repeatedly administered (i.g.) to female C57BL/6N mice prior to mating (Fig. 1, yellow box), before their male and female offspring were exposed to chronic psychosocial stress during adulthood. In males, chronic adult stress was induced by CSC (Fig. 1, blue box) [35, 47], while in females the SIP [48–50] was employed (Fig. 1, red boxes). As neither CSC nor SIP reliably enhanced general/social anxiety-related behavior in male and female offspring, making it difficult to interpret the intergenerational stress protective effects of *M. vaccae* ATCC 15483^T^ on these parameters, these results are reported in the supplementary part (Fig. S1; supplementary results/discussion section). All statistical analyses and effects are reported in Tab. S2.

### *M. vaccae* ATCC 15483^T^ intergenerationally protects against numerous negative consequences of chronic psychosocial stress in male offspring

CSC and/or SIP negatively affect physiology [29, 30, 35, 42, 51–57], the immune system [41, 53, 54], and long bone length [34, 51]. Moreover, rapid-growing mycobacteria are protective against many of these negative stress consequences within one generation [44, 58, 59]. Here, we examined whether *M. vaccae* ATCC 15483^T^ (i.g.) also intergenerationally protects against typical negative CSC and/or SIP effects.

#### Male offspring

Our data indicate that the CSC-induced increase in relative adrenal (Fig. 2a) and spleen (Fig. 2b) weight, decrease in relative thymus weight (Fig. 2c) and long bone length (Fig. 2d), as well as enhancement of splenic TLR2 (Fig. 2e) and TLR4 (Fig. 2f) protein levels were ameliorated/prevented in offspring of *M. vaccae* ATCC 15483^T^ vs. BBS dams. Moreover, CSC vs. SHC offspring of BBS but not *M. vaccae* ATCC 15483^T^ dams showed an increased basal and LPS-induced (Fig. 2g) as well as delta (LPS-basal; Fig. 2h) ex vivo cell viability of isolated splenocytes in the absence of corticosterone. In addition, compared with the 0 µM corticosterone condition, delta (LPS-basal; 0 µM corticosterone set to 100 %) ex vivo cell viability in CSC offspring of *M. vaccae* ATCC 15483^T^ but not BBS dams was not reduced in the presence of 0.1 µM (Fig. 2i), indicating that *M. vaccae* ATCC 15483^T^ intergenerationally protected against CSC-induced glucocorticoid resistance. Consistently, delta (LPS-basal; 0 µM corticosterone set to 100 %) ex vivo cell viability was lower in CSC offspring of *M. vaccae* ATCC 15483^T^- vs. BBS-administered dams at 5 µM corticosterone. Finally, offspring of dams administered with *M. vaccae* ATCC 15483^T^ but not BBS were also protected against the CSC-induced reduction in splenic CD4^+^CD25^+^FoxP3^+^ Tregs in percent of all splenocytes (Fig. 2j; Fig. S2a) as well as the calculated “Treg suppressive capacity per animal” (Fig. 2k; Fig. S2b-d).

In contrast, *M. vaccae* ATCC 15483^T^ intergenerationally was not protective against the CSC-induced increase in chemotactic activity of isolated white blood cells (Fig. 2l). Moreover, the percentages of splenic monocytes/MO-MDSCs (Fig. 2m), neutrophils (Fig. 2n) and PMN-MDSCs (Fig. 2o) in CSC vs. SHC offspring of both *M. vaccae* ATCC 15483^T^ and BBS dams were significantly or by trend (Fig. 2n; *M. vaccae* ATCC 15483 ^T^: *P* = 0.060) increased. Parameters, which were not affected by CSC in offspring of BBS dams are reported in the supplementary part (Figs. S3a-c; supplementary results/discussion section).

#### Female offspring

Neither *M. vaccae* ATCC 15483^T^ nor SIP affected any of the assessed readouts (Fig. S3d-t), except the SIP-induced increase in the percentage of splenic CD11b^+^Ly6C^+^Ly6G^–^ monocytes/MO-MDSCs, which was, as in males, not prevented by *M. vaccae* ATCC 15483^T^ (Fig. S3u).

### *M. vaccae* ATCC 15483^T^ intergenerationally diversifies male offspring’s fecal microbiome and increases the relative abundance of taxa known to produce short-chain fatty acids (SCFAs)

To determine possible mechanisms underlying the intergenerational stress-protective effects of *M. vaccae* ATCC 15483^T^ in male offspring, we next assessed their fecal microbiome composition prior to CSC. Permutational multivariate analysis of variance (PERMANOVA) on Bray-Curtis distance matrix (Fig. 3a) of the fecal microbiome revealed significant differences in β-diversity between offspring of *M. vaccae* ATCC 15483^T^ vs. BBS dams. Moreover, the number of observed ASVs (Fig. 3b), Shannon diversity index (Fig. 3c) and Chao1 index (Fig. 3d) were increased in offspring of *M. vaccae* ATCC 15483^T^ vs. BBS dams. As further Pielou’s evenness (Fig. 3e) was comparable between the groups, this effect seems to be driven by an increase in richness rather than evenness. Correlational analyses (Fig. 3f) revealed that the number of observed ASVs and the Chao1 index correlated significantly or by trend (*P* ≤ 0.1) negatively with parameters found to be increased by CSC in the BBS group (i.e., adrenal (Fig. 2a) and spleen weights (Fig. 2b), splenic monocytes/MO-MDSCs (Fig. 2m) and PMN-MDSC (Fig. 2o) counts, splenic TLR4 protein levels (Fig. 2f), basal splenic ex vivo cell viability in the absence of corticosterone (Fig. 2g) and delta (i.e., LPS-basal) splenic ex vivo cell viability in the presence of 0.1 µM and 5 µM corticosterone (Fig. 2i)) and positively with the majority of parameters found to be decreased by CSC in the BBS group (i.e., thymus weight (Fig. 2c), Treg counts (Fig. 2j), and Treg suppressive capacity per animal (Fig. 2k)). Administrations of *M. vaccae* ATCC 15483^T^ vs. BBS intergenerationally also altered the community composition of the fecal microbiome prior to CSC exposure (Fig. S4a) and increased the relative abundance of Oscillospirales [60], Clostridia vadin BB60 group [61], *Roseburia* [62, 63]. Further intergenerational effects of *M. vaccae* ATCC 15483^T^ on the community composition of offsprings’ fecal microbiome are reported in the supplementary part (supplementary results/discussion section; Fig. S4).

To determine whether the above reported changes in the fecal microbiome prior to CSC were stable during chronic stress exposure (i.e., stress-resistant), we further analyzed the fecal microbiome of CSC offspring of *M. vaccae* ATCC 15483^T^ and BBS dams after CSC exposure. PERMANOVA on Bray-Curtis distance matrix again revealed that CSC offspring of *M. vaccae* ATCC 15483^T^ vs. BBS dams differed with respect to β-diversity (Fig. 3g). Moreover, the number of observed ASVs (Fig. 3h), as well as Shannon diversity index (Fig. 3i) and Chao1 index (Fig. 3j), but not Pielou’s evenness (Fig. 3k), were higher in CSC offspring of *M. vaccae* ATCC 15483^T^ vs. BBS dams. Correlational analyses (Fig. 3l) further revealed that the number of observed ASVs and/or Chao1 index correlated (i.e., significantly or by trend; *P* ≤ 0.1) negatively with adrenal weight (Fig. 2a), splenic TLR2 and TLR4 protein levels (Fig. 2e, f), basal splenic ex vivo cell viability in the absence of corticosterone (Fig. 2g) and delta (i.e., LPS-basal) splenic ex vivo cell viability in the presence of 0.1 µM corticosterone (Fig. 2i), and positively with thymus weight (Fig. 2c), tibia length (Fig. 2d), Treg counts (Fig. 2j), and Treg suppressive capacity per animal (Fig. 2k). After CSC, male offspring of *M. vaccae* ATCC 15483^T^ vs. BBS dams further showed an increased relative abundance of Ruminococcaceae [60, 64, 65] (Fig. S4c), which positively correlated (Fig. S4d) with splenic Treg counts (Fig. 2j) and the Treg suppressive capacity per animal (Fig. 2k).

### *M. vaccae* ATCC 15483^T^ affects dams’ fecal microbiome

As maternal microbiota can shape offspring microbial colonization and immune development [66, 67], we additionally investigated whether repeated *M. vaccae* ATCC 15483^T^ administration to females prior to mating alters their fecal microbiome before mating, at the day of giving birth and at weaning. PERMANOVA on Bray-Curtis distance matrix revealed a significantly different fecal microbial β-diversity between the BBS and *M. vaccae* ATCC 15483^T^ group, both at mating and weaning (Fig. 4a). Moreover, lower numbers of observed ASVs (Fig. 4b) and Chao1 index (Fig. 4d) were found in *M. vaccae* ATCC 15483^T^ vs. BBS dams at weaning, while Shannon diversity index (Fig. 4c) and Pielou’s evenness (Fig. 4e) were comparable between the groups at all time points assessed.

Further effects on α-diversity and community composition of the fecal microbiome of dams are reported in the supplementary part (Fig. 4; Fig. S5h; supplementary results/discussion section).

### *M. vaccae* ATCC 15483^T^ does not affect maternal behavior and physiology/immunology

To determine whether the intergenerational effects of *M. vaccae* ATCC 15483^T^ on offspring’s fecal microbiome and stress resilience are associated with changes in maternal care or maternal physiology/immunology, we assessed litter characteristics as well as maternal behavior and physiology/immunology in *M. vaccae* ATCC 15483^T^ and BBS dams. As the groups did not differ in any of the assessed parameters, these results are reported in the supplementary part (Figs. S5a-g, S6; supplementary results/discussion section).

## Discussion

Our results show that repeated administration (i.g.) of a heat-killed preparation of *M. vaccae* ATCC 15483^T^ to female C57BL/6N mice prior to mating intergenerationally protected their male offspring against numerous negative consequences of chronic psychosocial stress (i.e., CSC). Moreover, fecal microbial community analysis in combination with correlational analyses support the hypothesis that *M. vaccae* ATCC 15483^T^ intergenerationally promotes stress resilience, at least in part, *via* diversifying the fecal microbiome and increasing the relative abundance of bacterial taxa known to be potent SCFA producers. The intergenerational stress-protective effects of *M. vaccae* ATCC 15483^T^ in females are difficult to interpret, as SIP did not reliably induce signs of chronic stress in female offspring.

### *M. vaccae* ATCC 15483^T^ intergenerationally protects male offspring against numerous negative consequences of chronic psychosocial stress

*M. vaccae* ATCC 15483^T^ (i.g.) intergenerationally protected against the CSC-induced increase in relative adrenal weight as well as the CSC-induced decreases in relative thymus weight and long bone length, which represent typical signs of chronic stress and have been reported earlier in CSC mice [34, 35, 47, 51, 52]. In contrast, repeated administration of *M. vaccae* NCTC 11659 prior to CSC exposure was not protective against CSC-induced adrenal enlargement [25, 30, 41]. Therefore, further studies are needed to explore whether i) intergenerational effects are stronger than intragenerational ones, or ii) the two closely related mycobacterial species differ in their mechanism of action.

In further support for rapid-growing mycobacteria to intergenerationally promote stress resilience, *M. vaccae* ATCC 15483^T^ prevented the CSC-induced upregulation of splenic TLR2 and TLR4 and the development of splenic functional ex vivo glucocorticoid resistance. The latter is in line with earlier studies reporting that *M. vaccae* NCTC 11659 administered prior to CSC has comparable effects [30]. Although the CSC-induced splenomegaly was less pronounced in male offspring of *M. vaccae* ATCC 15483^T^ vs. BBS dams, this seems not to be indicative for an intergenerational resilience effect of *M. vaccae* ATCC 15483^T^. As the relative spleen weight correlated positively with the bite score in all CSC-exposed males, this effect is rather due to slightly but not significantly lower bite wound severity in CSC offspring of *M. vaccae* ATCC 15483^T^ vs. BBS dams. Note, stress-induced spleen invasion of CD11b^+^ myeloid cells and, thus, development of splenomegaly is critically dependent on the occurrence of bite wounds during chronic psychosocial stressors allowing direct physical interaction of the conspecifics [55, 68, 69]. In line with these findings, repeated administrations (i.g.) of *M. vaccae* NCTC 11659 prior to CSC are neither protective against CSC-induced bone marrow myelopoiesis nor against the bite wound-dependent splenic invasion of myeloid cells and splenomegaly [30]. Consistent with the fact that *M. vaccae* ATCC 15483^T^ intergenerationally was not protective against the CSC-induced splenic invasion of myeloid cells and splenomegaly, it did also not prevent the well-known [53] CSC-induced increase in chemotactic activity of isolated white blood cells in male offspring.

Most strikingly, male offspring of dams administered with *M. vaccae* ATCC 15483^T^ were fully protected against the CSC-induced reduction in splenic CD4^+^CD25^+^FoxP3^+^ Tregs (i.e., in % of all splenocytes) and the decline in the calculated “Treg suppressive capacity per animal”. This is in line with our earlier studies reporting comparable findings in adult male mice receiving repeated administration (i.g.) of *M. vaccae* NCTC 11659 prior to CSC [30].

In female offspring, SIP only induced an increase in the percentage of splenic CD11b^+^Ly6C^+^Ly6G^–^ monocytes/MO-MDSCs, which was, as in males, not prevented by *M. vaccae* ATCC 15483^T^. In line with the latter, a SIP-associated, bite wound-independent (note that we did not observe any signs of bite wounding in females during SIP exposure) and *M. vaccae* NCTC 11659-insensitive splenic invasion of myeloid cells in female mice is in line with our recent study [41].

### *M. vaccae* ATCC 15483^T^ intergenerationally diversifies male offspring’s fecal microbiome and increases the relative abundance of taxa known to produce SCFAs

Prior to CSC, male offspring of *M. vaccae* ATCC 15483^T^ vs. BBS dams differed significantly in β-diversity of the fecal microbiome, suggesting the latter to be involved in mediating the intergenerational stress-protective effects of *M. vaccae* ATCC 15483^T^. In line with this hypothesis, *M. vaccae* ATCC 15483^T^ vs. BBS intergenerationally increased fecal α-diversity in male offspring prior to CSC. Higher α-diversity of the gut microbiota is often associated with increased health outcomes in human population studies [70–72]. An increased microbial α-diversity also has been reported in clinical [73, 74] and preclinical [75] studies as consequence of prebiotic administrations. In contrast, a decline in human fecal α-diversity has occurred during the course of evolution, particularly in urban areas of highly-industrialized countries [76–78]. Consistent with the hypothesis that the fecal microbiome plays a critical role in the intergenerational stress-protective effects of *M. vaccae* ATCC 15483^T^, correlational analyses revealed that several measures of α-diversity correlated negatively with parameters found to be increased by CSC in offspring of BBS dams and positively with the majority of parameters found to be decreased by CSC in this group.

After CSC, and this is in line with the above, male offspring of dams that had received repeated administration of *M. vaccae* ATCC 15483^T^ vs. BBS also differed with respect to β-diversity and showed an increased α-diversity. The latter is of particular importance, as stressor exposure has been shown to cause dysbiosis by impacting specific gut bacteria and producing community structure changes, including reductions in α-diversity [25, 79–82]. Again, an important role for the fecal microbiome in mediating the intergenerational stress-protective effects of *M. vaccae* ATCC 15483^T^ is suggested by correlational analyses. The latter revealed that the number of observed ASVs and/or Chao1 index assessed in CSC offspring of BBS and *M. vaccae* ATCC 15483^T^ dams correlated negatively with multiple parameters increased by CSC in offspring of BBS dams and positively with multiple parameters decreased by CSC in this group.

Importantly, ANCOM-BC2 analysis on the community composition of the fecal microbiome further support the hypothesis that the fecal microbiome plays a critical role in mediating the intergenerational stress-protective effects of *M. vaccae* ATCC 15483^T^. Briefly, repeated administration of *M. vaccae* ATCC 15483^T^ intergenerationally increased the relative abundances of taxa known to be potent SCFA producers and/or associated with health benefits when administered as probiotics, including Oscillospirales [60], Clostridia vadin BB60 group [61], *Roseburia* [62, 63], (all prior to CSC) and Ruminococcaceae [60, 64, 65] (after CSC). Strikingly, the relative abundance of all these SCFA-producing taxa correlated positively with Treg counts and Treg suppressive capacity per animal. Note, SCFAs regulate the size and function of the colonic Treg pool and protect against experimentally induced colitis in mice [83]. Butyrate, the SCFA highly produced by *Roseburia* [84], activates the receptor for niacin in the colon called Gpr109a, with dendritic cells and macrophages isolated from Gpr109a knockout mice showing a reduced capacity to promote Tregs even in the presence of butyrate [85, 86]. Butyrate further relieves histone H3 deacetylase (HDAC) inhibition of the *FoxP3* gene and promotes the formation of Tregs [86–89].

However, maternal exposure to pre-conception helminth infection has been shown to provide long-term, nursing-associated, and antibody-independent immune benefits to offspring, mediated by maternally derived pathogen-experienced Th2-competent CD4 T cells [90]. Therefore, it cannot be excluded at the moment that the intergenerational stress-protective effects of *M. vaccae* ATCC 15483^T^ are independent of changes in the fecal microbiome in male offspring of dams that received repeated administration of *M. vaccae* ATCC 15483^T^ [89, 91].

### *M. vaccae* ATCC 15483^T^ affects the fecal microbiome of the dams

While both groups were comparable at birth, the fecal microbiome of *M. vaccae* ATCC 15483^T^ vs. BBS dams differed in β-diversity at mating and in α- and β-diversity at weaning. In line with the latter and confirming that rapid-growing mycobacteria in general affect the fecal microbiome, our earlier findings show that male mice repeatedly administered (subcutaneously) with *M. vaccae* NCTC 11659 vs. BBS also differ in fecal microbial α- and β-diversity [25]. One explanation for the episodic effect of *M. vaccae* ATCC 15483^T^ on fecal microbial diversity in dams of the current study could be related to its effects on Tregs. Briefly, both *M. vaccae* NCTC 11659 and *M. vaccae* ATCC 15483^T^ have been shown earlier [25, 30] and in the current study to promote immunoregulation *via* positively affecting the Treg compartment in male mice. Thus, it is likely that *M. vaccae* ATCC 15483^T^ also in dams of the current study positively affected the Treg compartment. As an individual’s immune system and its microbiome are highly interconnected [92–95], these Treg-promoting effects of *M. vaccae* ATCC 15483^T^ in dams might have affected the fecal microbiome at mating and weaning. At birth no differences in fecal microbial diversity could be detected between the groups, as the female immune system is strongly affected during pregnancy, with immune responses particularly at the beginning of pregnancy being geared towards a reduction of T cells and an expansion of DCregs and Tregs [92, 95, 96]. As pregnancy-associated changes in the fecal microbiome have been hypothesized to drive changes in the Treg compartment [89, 91], further studies are warranted to unravel the detailed mechanisms.

### Possible mechanisms underlying the intergenerational effects of *M. vaccae* ATCC 15483^T^ on offspring’s fecal microbiome

As we did not reveal any differences in litter characteristics, maternal care or maternal physiology, these factors are unlikely as possible mediators for the intergenerational effects of *M. vaccae* ATCC 15483^T^ on offspring’s fecal microbiome.

Another possible mediator might be the dam’s milk, as immune components, including complement components, immune cells, regulatory cells, and antibodies are delivered *via* the milk from a dam to an infant [97, 98]. In line with the latter, it has been shown that colonization of adult germ-free postnatally stressed and control mice with the same microbiota produces distinct microbial profiles [94], which have been further detected in the progeny across two generations [99]. Although we have not assessed dam’s milk antibody concentrations or immune cell composition/counts, the complement system seems not to be involved in mediating the intergenerational effects of *M. vaccae* ATCC 15483^T^ on male offspring’s microbiome composition. The latter is indicated by the fact that C3 mRNA expression in breast tissue of BBS and *M. vaccae* ATCC 15483^T^ dams was comparable between the groups.

Another possible mediator for the intergenerational effects of *M. vaccae* ATCC 15483^T^ on offspring’s fecal microbiome might be the transfer of an affected microbiome itself [100, 101]. The latter hypothesis is in line with the fact that maternal gut microbes have been shown to be more persistent in the infant gut compared to those from other sources [102]. In addition, maternal high-fat diet-induced dysbiosis of the gut microbiome is vertically transmitted to F1 offspring [103–105]. Moreover, pre-pregnancy colonization of young dams with microbiome from aged female donors significantly and long-lastingly affected the gut microbial composition of their pups [106]. Given the coprophagic nature of mice [107, 108], and the fact that dams in the current study did not differ in the diversity of their fecal microbiome at birth, but at weaning (i.e., during lactation), vertical transmission of the *M. vaccae* ATCC 15483^T^-affected fecal microbiome from the dams to their male offspring is likely fecal-orally mediated. However, future cross-fostering studies would provide an elegant approach to investigate the underlying mechanisms in greater detail.

In summary, our data support the hypothesis that developing immunomodulatory approaches, such as repeated i.g. administrations with heat-inactivated immunoregulatory rapid-growing mycobacteria, have potential for facilitating resilience against chronic psychosocial stressors, even in an intergenerational manner. The latter is in line with epidemiological studies showing that prenatal exposure to biodiverse rural environments can beneficially program the offspring immunity [109, 110].

## Supplementary information

Supplementary Information

Figure S1

Figure S2

Figure S3

Figure S4

Figure S5

Figure S6

## Data Availability

The datasets generated and analysed in the current study are available from the corresponding author upon reasonable request.
