# Supplementary material for: Microbial Legacy: Mycobacterium vaccae ATCC 15483T intergenerationally diversifies the microbiome and enhances stress resilience in male mice
Source: Mol Psychiatry. 2026 May 22;31(10):5538–49. doi: 10.1038/s41380-026-03638-9 (PMC13569420; doi:10.1038/s41380-026-03638-9)
Supplement: Supplementary file 1 [file 41380_2026_3638_MOESM1_ESM.docx]

**Supplementary Information: Schiele et al., *Molecular Psychiatry***

**1. Supplementary Materials and Methods Section**

1.1 **Intragastric (i.g.) administration of *M. vaccae* ATCC 15483^T^ (MvacATCC) or its vehicle borate-buffered saline (BBS)**

Administrations (i.g.) were performed as described previously.^1^ Nulliparous female mice received either 100 µL MvacATCC (concentration: 1 mg/ mL; final dose of 0.1 mg) diluted in sterile BBS or 100 µL BBS (vehicle) via a 20-gauge gavage needle (FST, Cat. No.: 18060-20) on experimental days –21, –14 and –7 prior to mating. Of note, this is the same dose that our group has used previously for s.c. and i.g. administrations of *M. vaccae* NCTC 11659.^2^

1.2 **Maternal behavior**

Maternal behavior was observed daily throughout the first week following birth (postnatal days 1 – 6, except on postnatal day 3) as described previously with slight modifications.^3^ In the morning between 0700 – 1000 h, maternal “on nest” behavior including arched-back or blanket posture nursing, licking, grooming or carrying of pups, nest building, self-grooming and sleeping was assessed in four observation intervals (5 sec each) per 10 min. Documented “on nest” behavior was calculated as number of observation intervals in percent of total number of observation intervals (resulting in 72 intervals in 3 h) set to 100 %. Note, two MvacATCC dams were excluded due to loss of the litters between birth and weaning.

1.3 **Pup retrieval test in dams**

All dams were tested for maternal responsiveness on postnatal day 3 using the pup retrieval test based on previously published test procedures.^4, 5^ Briefly, the litters were separated from their dams and placed in boxes on top of warm mats for 60 min. For the pup retrieval test, the bulk of nesting material was removed. Following 60 min of separation, a maximum of six pups were distributed along the walls and in the center of the home cage, before the respective dam was placed back into the cage onto the position of the original nest. The test procedure was recorded for 15 min for the assessment of the latency to retrieve first pup (sec) and the latency to place all pups back into the nest (sec). Following the end of the pup retrieval test, the nest material and all (remaining) pups were placed back in the nest of the home cage. Of note, one BBS-dam was excluded from the parameters “latency to retrieve first pup” and “latency to place all pups back into the nest” as it did not perform. Additionally, two BBS dams were excluded from the parameter “latency to place all pups back into the nest” because they did not retrieve all six pups during the testing period and one MvacATCC dam was excluded from the parameter “latency to place all pups back into the nest” as the litter size was smaller than *N* = 6 (pups: *N* = 3).

1.4 **Open-field/ novel object test**

To test for general anxiety-related behavior, the open-field / novel object test^6, 7^ was performed as previously published.^2^ Briefly, during open field exposure, the test arena (45 cm length × 27 cm width × 27 cm height; 350 lx) was subdivided into an inner (9 × 27 cm) and an outer zone. The mouse was placed into the inner zone and allowed to explore the arena for 5 min. After 5 min of open field exploration, a round plastic object (diameter: 3.5 cm; height: 1.5 cm) was placed in the middle of the arena and the mouse was allowed to explore the arena containing the unfamiliar novel object for 5 min. During open field exploration, the overall distance moved was analyzed as an indicator of general locomotion. Moreover, as an indicator of anxiety-related behavior, the entries into the inner zone were counted and the time in the inner zone was recorded. During novel object exploration, the entries into the direct contact zone containing the novel object and the time in the contact zone were analyzed. The test arena was cleaned thoroughly with water before each test. All parameters were analyzed using EthoVision XT (v11.5.1022; Noldus Information Technology, Wageningen, The Netherlands). Of note, one MvacATCC-GHC female of Experiment 1 in the novel object condition was excluded from the analysis due to jumping out of the arena during recording.

**1.5 Social preference/ avoidance test**

General and social anxiety-related behavior was assessed employing the social preference/ avoidance test as previously described^2, 8^ with minor modifications. Briefly, the experimental mouse was placed in the social preference/ avoidance test box (45 cm length × 27 cm width × 27 cm height; 20 lx) for 30 sec to habituate to the environment before a small empty wire mesh cage (8.5 cm length × 7.5 cm width × 6.5 cm height) was introduced for 150 sec (empty condition). This initial period informs about general anxiety-related behavior, as the mouse is exposed to a novel object. Afterwards, the empty cage was exchanged with an identical cage containing an unfamiliar male (when testing males) or female (when testing females) C57BL/6N mouse respectively for another 150 sec (social condition). Time spent in the direct contact zone around the wire mash cage was recorded using EthoVision XT during both 150 s trials. To assess social preference, time spent in the direct contact zone during “social” cage exposure was divided by the sum of the total time spent in the direct contact zone (during “empty” + “social” cage exposure). The box was cleaned thoroughly with water before each test.

1.6 **Chronic subordinate colony housing (CSC) paradigm**

The chronic subordinate colony housing (CSC) paradigm was conducted as previously described.^2^ Briefly, mice were weighed and assigned either to the single-housed control (SHC) condition or to the CSC group, according to their body weight. To induce chronic psychosocial stress, four experimental male C57BL/6N mice were co-housed together with a dominant male CD-1 mouse for 21 consecutive days. To avoid habituation, CSC mice were transferred to the home cage of a novel dominant aggressor CD-1 mouse on days 8 and 15 of CSC. Body weight and possible bite wounds were assessed twice per week. Before the beginning of the CSC procedure, all potential dominant CD-1 mice were tested for their aggressiveness to exclude individuals severely injuring their conspecifics. To confirm the intended dominant/subordinate hierarchy within each colony, mice were always videotaped during the first hour after colony formation (10:00 – 11:00 a.m.) on days 1, 8 and 15 of CSC. SHC mice remained undisturbed in their home cages except for changing their bedding once a week. In a previous study, we, but also others, convincingly demonstrated, that single housing is the adequate control group for the CSC paradigm, as group housing itself was shown to be stressful and to affect parameters assessed routinely in studies employing the CSC paradigm.^13, 14^

1.7 **Social instability paradigm (SIP)**

To induce chronic psychosocial stress in C57BL/6N female mice, the SIP was conducted as published previously.^8^ Briefly, the cage composition of experimental mice was changed every third day over a period of seven consecutive weeks. To minimize the chance of repeatedly encountering the same cage mates, group rotation was randomized using GraphPad “Assign subjects to groups” (GraphPad Software, San Diego, CA) online version. Body weight and occurring bite wounds were assessed every time the cage composition was changed. Respective group-housed controls (GHCs) were housed in groups of 3 - 4 mice for 7 weeks.

1.8 **Bite wound severity assessment**

To assess the severity of bite wounds in CSC mice, a bite score established by our group was used as previously described.^15^ Briefly, the skin (with fur attached) of the CSC mice was removed so that skin and body were still connected at the tail root and the legs. Afterwards pictures depicting both skin (dermal) and body (subdermal) of all CSC mice were taken using identical camera position and settings. All pictures were digitally overlaid with a standardized grid consisting of 20 squares (4 × 5 squares; each square 180 × 240 pixels; overall 720 × 1200 pixels) covering the skin and 20 squares (4 × 5 squares; each square 180 × 240 pixels; overall 720 × 1200 pixels) covering the body. Subsequently, the severity of the received bite wounds was scored according to a developed bite score, considering both size and intensity of wounds on the skin (dermal) and on the body (subdermal): body – affected area: 0, no bites; 1, <1/3 of the area; 2, 1/3–2/3 of the area; 3 > 2/3 of the area; maximum subscore: 60. Body – intensity of injuries: 1, mild; 2, severe; maximum subscore: 40. Skin – affected area: 0, no bites; 1, single bites; 2, several bites; 3 spacious bites; 4, bites in the whole square; maximum subscore: 80. Skin – intensity of bite wounds: 1, mild; 2, moderate; 3, severe; 4, necrotic; maximum subscore: 80. Skin – degree of purulence: 0, no purulence; 1, mild purulence; 2, severe purulence; maximum subscore: 40. The total bite score of each mouse represents the sum of the skin (dermal) and the body (subdermal) and possible scores range from 0 to 300. Bite score assessment was performed by an investigator blinded to the group distribution.

1.9 **Assessment of chemotactic activity of neutrophil/ polymorphonuclear (PMN) – myeloid-derived suppressor cells (MDSCs) – enriched white blood cells**

The chemotactic assay of neutrophil/ PMN-MDSC-enriched white blood cells was performed as described previously.^1^ Briefly, one aliquot of trunk blood was collected in EDTA-coated tubes (Sarstedt; Nümbrecht, Germany). Plasma was partially removed from EDTA blood following gentle centrifugation (400 × g, 5 min, RT). Remaining EDTA cruor was gently resuspended in NaCl (0.9 %) and pooled per experimental group before being loaded on top of a Ficoll (GE Healthcare, Chicago, IL, USA) layer and centrifuged (400 × g for 30 min, RT; lowest level of acceleration, no deceleration). After removing the supernatant (containing remaining plasma, mononuclear cells and Ficoll layer), the pellet was resuspended in NaCl (0.9 %) before being mixed with dextran (Sigma Aldrich, St. Louis, MO, USA; 5 % in NaCl). Following a 45 min step of dextran sedimentation, the supernatant was transferred to a new tube, and centrifuged (400 × g for 5 min, RT). After discarding the supernatant, erythrocyte lysis was performed with Ampuwa H_2_O (Fresenius, Bad Homburg, Germany) and was stopped with 2.7 % NaCl. Following a final step of centrifugation (400 × g for 5 min, RT), the cells were resuspended in PBS (Gibco, Carlsbad, CA, USA) + 0.1 % BSA and counted using a cell counter (ViCell XR, Beckman Coulter, Krefeld, Germany). Afterwards, cells were adjusted to a concentration of 8 × 10^4^ cells/ 44 µl, labelled with 2’,7’-bis-(2-carboxyethyl)-5-(6)-carboxyfluorescein (BCECF) for 30 min at 37 °C, centrifuged and resuspended in Hanks’ Balanced Salt Solution (HBSS; Gibco, Carlsbad, CA, USA) + 0.1 % BSA. The wells of the lower chamber of a 96-well chemotaxis mini-chamber (Neuroprobe, Cabin John, MD, USA) were loaded with 33 µl of *N*-formylmethionine-leucyl-phenylalanine (fMLP; 10^-2^ M stock solution diluted to 10^-6^ M working solution) as chemoattractant and separated from the upper chamber by a polycarbonate membrane (3 µm porosity; Neuroprobe). Cells (6 technical replicates per group, each with 44 µl) were then loaded into the wells of the upper chambers before incubating the plate for 30 min at 37 °C. After removing the membrane from the chamber, chemotactic activity was determined by measuring the fluorescence of the migrated cells on the membrane using a plate reader (Fluostar Optima, BMG Labtech GmbH, Offenburg, Germany) and the respective OPTIMA Software (v2.20R2). As Experiment 1 was conducted in two rounds, experimental groups of round 2 were adjusted to round 1 by a factor calculated out of the mean of the six technical replicates of the MvacATCC-SHC group of rounds 1 and 2.

1.10 ***Ex vivo* splenocyte isolation**

Splenocytes were isolated as previously described.^2^ Briefly, following homogenization of the spleens, erythrocytes were removed from the single-cell suspension by 2-min incubation with lysis buffer (155 mM NH_4_Cl, 10 mM KHCO_3_, 10 mM EDTA). The lysis was stopped by addition of HBSS containing 10 % heat-inactivated fetal calf serum (FCS). Afterwards, the cell suspension was passed through a 70 µm nylon cell strainer and washed, followed by assessment of the number of isolated splenocytes by use of a cell counter (ViCell XR, Bio-Rad Laboratories, Munich, Germany). Then, cells were suspended at a concentration of 5 × 10^6^ cells/ mL in RPMI containing 10 % FCS, 50 U/ mL penicillin and 50 µg/ mL streptomycin (RPMI supplemented (sup.)). Cells were then used for splenic glucocorticoid (GC) sensitivity assay. Remaining cells were either dissolved in freezing medium containing FCS and dimethyl sulfoxide (DMSO; C_2_H_6_OS Carl Roth GmbH + Co. KG, Karlsruhe, Germany; diluted 1:10) and frozen at –80 °C for later flow cytometric analysis (Section 1.16) or pelleted and snap frozen at –80°C for later protein isolation (Section 1.19).

1.11 **Preparation of cell-protected frozen splenocytes for flow cytometry**

Following complete thawing, splenocytes were dissolved in RPMI sup. and centrifuged. Following one wash step with RPMI sup. the cell count was assessed using an automated cell counter (ViCell XR, Beckman Coulter, Krefeld, Germany). Samples were then adjusted to a concentration of 5 x 10^6^ cells/ml with the appropriate volume of RPMI + 10% FCS + penicillin/ streptomycin added respectively to each sample.

1.12 **Flow cytometry analysis of splenic myeloid subpopulations**

Splenocytes were analyzed for the percentages of CD11b^+^Ly6C^+^Ly6G^–^ monocytes (MO)/ MO – myeloid derived suppressor cells (MDSCs), CD11b^+^Ly6C^–^Ly6G^+^ neutrophils and CD11b^+^Ly6C^+^Ly6G^+^ polymorphonuclear (PMN) – MDSCs as previously published.^2^ Briefly, a total of 1 × 10^6^ splenocytes were incubated with fluorescently labelled antibodies (CD11b, APC-eFluor 780, BD Bioscience, Franklin Lakes, NJ, USA; Ly6C, APC, BD Bioscience, Franklin Lakes, NJ, USA; Ly6G, V450, BD Bioscience, Franklin Lakes, NJ, USA) at 4 °C for 30 min. Following antibody incubation, cells were washed once with flow-associated cell sorting buffer (PBS, 10 % FCS, 0.1 % NaN_3_) before being fixated using the Foxp3/ Transcription Factor Staining Buffer Set Kit (eBioscience, Carlsbad, CA, USA). Another two washing steps with flow-associated cell sorting buffer were performed. Subsequently, samples were transferred into flow-associated cell sorting buffer and analyzed using a LSR II flow cytometer (BD Bioscience, Heidelberg, Germany) and Flow Jo Version 10 (Tree Star, Ashland, OR, USA).

1.13 **Flow cytometry analysis of splenic regulatory T cells**

Splenocytes were analyzed for the percentages of CD4^+^CD25^+^FoxP3^+^ regulatory T cells (Tregs) as previously described.^2^ Briefly, a total of 1 × 10^6^ splenocytes were incubated with fluorescently labelled antibodies for CD4 (clone RM4-5, APC eFluor-780, Thermo Fisher Scientific, Waltham, MA, USA) and CD25 (clone PC61.5, Thermo Fisher Scientific, Waltham, MA, USA) at 4 °C for 30 min. Following antibody incubation, cells were washed once with flow-associated cell sorting buffer (PBS, 10 % FCS and 0.1 % NaN_3_) before being fixated using the FoxP3 Transcription Factor Staining Buffer Set Kit (eBioscience, Carlsbad, CA, USA). Afterwards, the cells were incubated with an antibody for the intracellular marker forkhead-box protein P3 (FoxP3; clone FJK-16s, Thermo Fisher Scientific, Waltham, MA, USA) at 4 °C for 30 min. Subsequently, the cells were washed and resuspended in flow-associated cell sorting buffer. Flow cytometry samples were analyzed using a LSR II flow cytometer (BD, Biosciences, Heidelberg, Germany) and FlowJo Version 10 (Tree Star, Ashland, OR, USA).

1.14 **Protein isolation and western blot**

To assess splenic expression level of toll-like receptor (TLR) 4 and TLR2, protein isolation was performed from *ex vivo* isolated splenocytes followed by western blot analysis as described previously^2^ with minor modifications. Briefly, splenocytes were resuspended in an appropriate amount of cell extraction buffer (CEB; 1 mL/ 10^8^ cells; Life Technologies, Carlsbad, CA, USA) supplemented with complete mini protease inhibitor (Roche Diagnostics, Mannheim, Germany). After 30 min of incubation on ice, cell suspensions were centrifuged and the supernatant was collected and stored at –80 °C. Total protein concentrations were determined using a commercial kit (Bicinchoninic Acid Protein Assay Kit, Thermo Scientific, Rockford, USA). Equal amounts of protein lysates (15 µg) from each experimental mouse were loaded on a 4–20 % Mini-PROTEAN TGX Stain-Free Gel (Bio-Rad Laboratories, Munich, Germany). The amount of whole protein was detected using a Molecular Imager® ChemiDoc™ MP system (Bio-Rad Laboratories, Munich, Germany). Whole protein was then blotted on nitrocellulose blotting membranes (GE Healthcare Life Sciences, Germany). Membranes were blocked with 5 % bovine serum albumin (BSA) in Tris-buffered saline containing Tween (TBS-T). Membranes were then incubated with primary antibody for mouse anti-TLR4 (1:1000, Santa Cruz Biotechnology, Dallas, TX, USA) on a shaker over night at 4 °C. After incubation with HRP-conjugated goat anti-mouse secondary antibody (1:10000, Jackson Immuno Research, Cambridgeshire, UK) for 1 h at RT, membranes were developed using Clarity^TM^ Western ECL Substrate (Bio-Rad Laboratories, Munich, Germany). The membranes were stripped using re-Blot Plus Antibody Stripping Solution (Millipore GmbH, Schwalbach, Germany) and blocked with 5 % milk powder (MP) in TBS-T and were incubated with primary antibody for goat anti-TLR2 (1:200, R&D Systems, Minneapolis, MN, USA) on a shaker over night at 4 °C. After incubation with HRP-conjugated chicken anti-goat secondary antibody (1:1000, R&D Systems) for 1 h at RT, membranes were developed using Clarity^TM^ Western ECL Substrate (Bio-Rad Laboratories, Munich, Germany). Antibody binding was visualized on Molecular Imager® ChemiDoc™ MP system (Bio-Rad Laboratories, München, Germany). Semiquantitative densitometric analyses of the signals were performed using Image Lab^TM^ Software (BioRad Laboratories, Munich, Germany). TLR4 (~100 kDa) and TLR2 (~96 kDa) protein expression for each mouse was normalized to the amount of whole protein. Of note, for TLR2 as well for TLR4 protein expression analysis, two females (MvacATCC-GHC group) from Experiment 1 were excluded due to technical issues, respectively.

1.15 **Functional *ex vivo* glucocorticoid (GC) sensitivity assay of isolated splenocytes**

Freshly isolated splenocytes were stimulated with lipopolysaccharide (LPS) and CORT to assess functional *ex vivo* GC sensitivity as previously described.^2^ Briefly, isolated splenocytes (2.5 x 10^5^ cells/ well) were stimulated with LPS (*Escherichia coli* O111:B4; final concentration of 1.0 µg/ml, Sigma-Aldrich, Deisenhofen, Germany) or remained untreated to assess background activity (basal). For determination of GC sensitivity, unstimulated and LPS-stimulated cells were treated with different CORT (Sigma-Aldrich, Deisenhofen, Germany) concentrations (final concentrations: 0 µM, 0.1 µM and 5 µM) diluted in 95% ethanol. Cells were stimulated in flat bottom 96-well plates (final volume 100 µl/ well) and incubated for 24 h (37 °C, 5% CO_2_). Afterwards, cell viability was measured by a commercially available colorimetric assay (CellTiter 96 Aqueous One Solution Cell Proliferation Assay, Promega, Madison, WI, USA) and the absorbance (optical density (OD)) of each well was measured at 450 nm using an enzyme-linked immunosorbent assay (ELISA) plate reader (Fluostar Optima, BMG Labtech GmbH, Offenburg, Germany). All values were blank corrected. The delta cell viability was calculated by subtracting the OD of unstimulated cells from the corresponding LPS-stimulated well. Additionally, the resulting delta OD was set to 100% for the 0 µM CORT condition. A significantly lower delta cell viability of isolated splenocytes in the presence of CORT indicates a high GC sensitivity, while a high delta cell viability in the presence of CORT indicates a low GC sensitivity. A significantly increased delta cell viability of isolated splenocytes in the presence of CORT, independent of the dimension of the effect, is referred to as GC resistance. Of note, one female (BBS-GHC group) and one female (MvacATCC-SIP) of Experiment 1 were excluded from the analysis of splenic functional GC resistance (delta cell viability with CORT 0 µM set to 100%) due to technical issues.

1.16 **Treg suppression assay**

To investigate suppressive capacity of Tregs, CD4^+^CD25^+^ T cells were isolated and incubated for 48 hours with carboxyfluorescein succinimidyl ester-stained responder cells in the presence or absence of anti-CD3/28 before cell proliferation was measured via flow cytometry. In order to assess the suppressive capacity of Tregs, CD4^+^CD25^+^ regulatory T-cells were isolated and incubated with “responder splenocytes” (Note, Experiment 1 was conducted in two rounds; in round 1 a pool of isolated splenocytes from the respective male or female control group was used, while in round 2 splenocytes were isolated from a non-experimental male or female breeding mouse). Isolation and counting of responder splenocytes were performed as described in section 1.15 of Supplementary Material and Methods section. Following the adjustment to a concentration of 5 x 10^6^ cells/ml, the splenocytes were stained with CFSE according to the manufacturers protocol (CellTrace^TM^ CFSE Cell Proliferation Kit for flow cytometry; Invitrogen, USA). Subsequently, CFSE-stained splenocytes were automatically counted using a ViCell XR cell counter (Beckman Coulter, Krefeld, Germany) and adjusted to 4 x 10^6^ cells/ml in RPMI (RPMI-1640, Sigma-Aldrich, St. Louis, MO, USA; supplemented with FBS, Gibco®, Thermo Fisher Scientific, Waltham, MA, USA; penicillin and streptomycin, Gibco®, Thermo Fisher; ß-Mercaptoethanol (0.01%), Gibco®).

*Magnetic-activated cell sorting separation of CD4^+^CD25^+^ activated T cells*

To isolate CD4^+^CD25^+^ activated T cells from single cell suspensions of mouse splenocytes, the CD4^+^CD25^+^ Regulatory T Cell Isolation Kit for magnetic-activated cell sorting separation was used according to the manufacturer protocol with minor modification (Miltenyi Biotec Inc. (San Jose, CA, USA). CD4^+^CD25^+^ Tregs were isolated under sterile conditions followed by automatic cell counting (Vi-CELL® XR Cell Viability Analyzer (Beckman Coulter, Brea, CA, USA)) and adjustment to 1.2 x 10^6^ cell/ mL.

*Treg suppression assay and flow cytometry of CFSE-stained, isolated splenocytes*

The assay was adapted from previous literature.^17^ Three different conditions were plated as six technical replicates each in a round-bottom 96-well plate: basal, NoTreg and Treg condition. Basal condition: responder cells (2 x 10^5^ cells/ well); NoTreg condition: responder cells (2 x 10^5^ cells/ well) + anti-cluster of differentiations (aCD) 3/28 (100 µl per well, respectively); Treg condition: responder cells (2x 10^5^ cells/ well) + aCD3/28 + magnetic-activated cell sorting-separated CD4^+^CD25^+^ splenocytes (6x 10^4^ cells/ well). Therefore, the wells of the NoTreg and Treg conditions were pre-coated with aCD3 (Thermo Fisher Scientific, Inc., Waltham, MA, USA) at least 24 h prior to the experiment. Shortly before the plating of responder cells and CD4^+^CD25^+^ splenocytes, wells were washed once with PBS (Gibco life Technologies, United Kingdom) and aCD28 (Thermo Fisher Scientific, Inc.) was added to the wells. Plated cells were incubated for 48 h (37 °C, 5% CO_2_) centrifuged, resuspended in flow-associated cell sorting buffer (PBS, 10% FCS, 0.1% NaN_3_) and transferred into flow-associated cell sorting tubes before measuring cell proliferation using flow cytometry. To measure the suppressive capacity of isolated (magnetic-activated cell sorting of pooled spleens per group; 6 x 10^4^ cells in 6 technical replicates per group; for details see above) CD4^+^CD25^+^ T cells, the number of aCD3/28-stimulated proliferating CFSE^+^ responder cells in the presence of these CD4^+^CD25^+^ T cells was calculated in percentage of aCD3/28-stimulated proliferating CFSE^+^ responder cells in the absence of CD4^+^CD25^+^ T cells. To account for these differences in the percentage of CD4^+^CD25^+^FoxP3^+^ Tregs within the total splenocytes, a “Treg suppressive capacity per animal” was calculated according to the following formula: [(1/ mean of proliferating cells as technical replicates per group)/ percentage of FoxP3^+^ Tregs within CD4^+^CD25^+^ cells per animal] * percentage of CD4^+^CD25^+^FoxP3^+^ Tregs.

1.17 **Preparation for Ultra-High-Throughput Microbial Community Analysis**

Microbial community analysis was performed in fecal samples from male offspring of Experiment 1 collected before and after the CSC exposure), as well as from BBS and MvacATCC-treated dams of Experiment 1 (collected at time points “mating start”, “birth” and “weaning”). Briefly, at each day of sample collection, fecal samples were frozen and stored at –80 °C until DNA extraction was performed using the PowerSoil DNA extraction kit (MoBio Laboratories, Carlsbad, CA, USA) according to the manufacturer's instructions. For 16S rRNA gene amplicon sequencing and bioinformatics analysis, samples were shipped to Novogene GmbH (Munich, Germany). 16S rRNA gene amplicon sequencing was performed according to the in-house protocols of Novogene. Briefly, marker genes in isolated DNA were PCR-amplified targeting the V4 hypervariable region of the 16S rRNA gene modified with a unique identifier for each sample. All PCR reactions were carried out with 15 μL of Phusion® High -Fidelity PCR Master Mix (New England Biolabs); 2 μM of forward and reverse primers, and about 10 ng template DNA. Thermal cycling consisted of initial denaturation at 98 ℃ for 1 min, followed by 30 cycles of denaturation at 98 ℃ for 10 s, annealing at 50 ℃ for 30 s, and elongation at 72 ℃ for 30 s and 72 ℃ for 5 min. Afterwards the same volume of 1X loading buffer (contained SYB green) was mixed with PCR products and electrophoresis was operated on 2% agarose gel for detection. PCR products were mixed in equidensity ratios. Then, mixture PCR products were purified with Qiagen Gel Extraction Kit (Qiagen, Germany). Sequencing libraries were generated using TruSeq® DNA PCR-Free Sample Preparation Kit (Illumina, San Diego, CA, USA) following manufacturer's recommendations and indexes were added. The library was checked with Qubit and real-time PCR for quantification and bioanalyzer for size distribution detection. Quantified libraries were pooled and sequenced on the Illumina platform. Of note, as one BBS dam at the time point "birth" had to be excluded due to failing the quality control step, this animal was excluded from statistical analyses at all time points.

Bioinformatics analysis was performed according to the Novogene in-house protocols. Raw sequence data were demultiplexed and quality filtered to identify all observed amplicon sequence variants (ASVs). Forward and reverse reads were trimmed and truncated based on sequence quality. Diversity analyses were executed within QIIME2, with data rarefied to an even sampling depth of 34,861 reads per sample. Alpha‐diversity metrics [i.e., Pielou’s evenness, Shannon diversity index, Chao1, observed amplicon sequence variants (ASVs) were evaluated after samples were rarefied. Bray-Curtis distance matrix was used to assess differences in phylogenetic community composition (beta diversity), which was analyzed via a permutational multivariate analysis of variance (PERMANOVA) using the Adonis2 function in R (vegan package). Differential abundance analysis was conducted using the R package **ANCOM-BC2** (Analysis of Compositions of Microbiomes with Bias Correction 2). This method accounts for the compositional nature of microbiome data and sample-specific biases through an Expectation-Maximization (E-M) algorithm. Statistical significance at the taxon level was determined using Holm-Bonferroni adjusted p-values (q-values). The estimated log fold change (lfc) was used to assess both the direction and magnitude of differential abundance. In all ANCOM-BC2 analyses, taxa assigned as “unclassified” were excluded prior to statistical testing. Only significant differences in taxa that were above the established cut-off for microbial abundance analyses (0.1%) in the majority of samples were considered.

1.18 **Mammary gland tissue homogenization and assessment of C3 mRNA expression**

Mammary gland tissue was freed from hair using a scalpel before 10 mg of tissue were added to 350 µl RLT buffer containing 1 % ß-Mercaptoethanol in lysis tubes (innuSPEED Lysis Tube P, Innuscreen GmbH). Tubes were placed in a homogenizer (SpeedMill) and samples were homogenized using the program “soft tissue 2”. RNA isolation and RT-qPCR were conducted as previously described. ^18^ Mammary gland tissue homogenates were centrifuged at 300 g for 5 min at 4 °C and RNA was isolated according to the manufacturer’s (Qiagen Qiashredder™, Hilden, Germany) protocol. The RNA yield was quantified with a Qubit 2.0 fluorometer using the Qubit RNA BR Assay Kit (Thermo Fisher Scientific). cDNA was generated using the AffinityScript qPCR cDNA synthesis kit (Agilent Technologies) with oligo(dT) primers and stored at −80 °C until further use. RT-qPCR was performed using a qPCR cycler Mx3000P (Agilent Technologies) and Brilliant III Ultra-Fast SYBR Green QPCR Master Mix (Agilent Technologies). Primers were purchased from Biomer.net (Ulm, Germany) and Qiagen (Hilde, Germany). Relative gene expression was determined by the 2−DD Ct method^19^ and results are reported as fold change compared to controls. The primers used for real-time PCRs were complement C3 provided by QIAGEN (Cat. nr.: QT001109270) and as housekeeping gene HPRT1 (Biomers, self-designed).

1.19 **Statistical Analysis**

For statistical analysis and graphical illustrations of data not referred to in the section about microbiome analysis, GraphPad Prism (version 10.1.1, GraphPad Software, LCC), was used as described previously.^2^ Briefly, Kolmogorov-Smirnov test with Lilliefors’ correction was employed to test for normal distribution. Normally distributed data sets were tested for one extreme outlier per group employing Grubbs test.^20^ The outliers were excluded from further analysis. Normally distributed data sets were analyzed by parametric statistics, i.e., two-tailed Student’s *t*-test (one factor, two independent samples, with Welch’s correction when appropriate), two-way analysis of variance (ANOVA; two factors, two or more independent samples) and Linear-Mixed-Model (LMM; more than one factor, more than two dependent samples). Additionally, effect sizes (η^2^) for all normally distributed behavioral data are included in Table (Tab.) S2. Non-normally distributed data sets were analyzed by non-parametric statistics, i.e. Mann-Whitney *U* test (MWU; one factor, two independent samples), Wilcoxon matched-pairs signed rank test (one factor, two dependent samples) and Friedman ANOVA (one factor, more than two dependent samples). All statistical tests comparing more than two samples were followed by *post hoc* analysis using Bonferroni pairwise comparison (parametric statistics) or Dunn’s multiple comparison (non-parametric statistics), when a significant main effect was found. Data are presented as mean + SEM including individual values (black dots). The level of significance was set at *P* ≤ 0.05. A detailed summary of all statistical tests performed, exclusion of extreme outliers and the respective results including *P*-values can be found in Tab. S2 in the supplementary information.

**2. Supplementary Results/Discussion Section**

***M. vaccae* ATCC 15483^T^ intergenerationally protects against mild negative behavioral consequences of chronic psychosocial stress in male and female offspring.**

To investigate whether administering *M. vaccae* ATCC 15483^T^ to female mice prior to mating affects their offsprings’ behavior or protects their offspring against repeatedly reported negative behavioral consequences of CSC^7, 9, 21^ or SIP^22^, male and female offspring were tested before and after chronic adult stress exposure in the social preference/ avoidance test and the open-field/ novel object test, respectively. Importantly, *M. vaccae* ATCC 15483^T^ administered (i.g.) repeatedly to females prior to mating did not negatively affect social (Fig. S1a, b) or general (Fig. S1c-l) anxiety-related behavior in male (Fig. S1a, c-g) and female (Fig. S1b, h-l) offspring. However, *M. vaccae* ATCC 15483^T^ (i.g.) intergenerationally prevented the mild CSC-induced increase in general anxiety-related behavior in male offspring. The latter was indicated by a lower number of entries into the contact zone during novel object exploration^6^ in CSC offspring of BBS dams compared with both SHC offspring of BBS dams and CSC offspring of *M. vaccae* ATCC 15483^T^ dams (Fig. S1f). Moreover, SIP female offspring of BBS- but not *M. vaccae* ATCC 15483^T^-administered dams travelled more during open-field exploration (Fig. S1h) when compared with respective GHC offspring.

***M. vaccae* ATCC 15483^T^ intergenerationally protects against numerous negative consequences of chronic psychosocial stress in male offspring.**

The percentages of splenic monocytes/ MO-MDSCs (Fig. 2m) as well as LPS-induced (Fig. 2g) and delta (LPS-basal; Fig. 2h) *ex vivo* splenocyte cell viability were increased in SHC male offspring of *M. vaccae* ATCC 15483^T^ vs. BBS dams. The latter suggests that *M. vaccae* ATCC 15483^T^ intergenerationally has adjuvant-like effects on the immune system, as it has been reported in mice directly administered (subcutaneously or i.g.) with rapid-growing mycobacteria.^2, 8, 23^ The bite score between both CSC groups (BBS: 47.93 + 11.00, *M. vaccae* ATCC 15483^T^: 35.00 + 5.938; mean + SEM; MWU) was statistically not different and positively associated (rho = 0.430; *P* = 0.034) with the relative spleen weight.

Moreover, CSC in male offspring of BBS dams did neither affect the capacity of isolated CD4^+^CD25^+^ splenocytes to suppress the anti-CD3/CD28-induced proliferation of carboxyfluorescein succinimidyl ester-labeled whole splenocytes (responder cells; Fig. S2b-d; Fig. S3a, b), nor the percentage of splenic FoxP3^+^ cells within the CD4^+^CD25^+^ subset (Fig. S3c). Therefore, the strong CSC-induced decline in the calculated "Treg suppressive capacity per animal" in male offspring of BBS dams in the current study (Fig. 2k) seems to be mediated exclusively by a reduced percentage (Fig. 2j) but not function (Fig. S3b) of Tregs.

Interestingly, SHC male offspring of *M. vaccae* ATCC 15483^T^ vs. BSS dams showed a reduced capacity to suppress stimulated carboxyfluorescein succinimidyl ester-labeled responder cells (Fig. S3b), despite the finding that the percentage of splenic FoxP3^+^ cells within the CD4^+^CD25^+^ subset was even increased (Fig. S3c; main effect for factor treatment, Tab. S2). The latter effects were both more pronounced in respective CSC vs. SHC mice (Fig. S3b, c). Together with the finding that *M. vaccae* ATCC 15483^T^ intergenerationally protects against the CSC-induced decline in splenic CD4^+^CD25^+^FoxP3^+^ Tregs (in % of all splenocytes), the latter supports the hypothesis that the protective effect of *M. vaccae* ATCC 15483^T^ against the CSC-induced decline in the calculated "Treg suppressive capacity per animal" is mediated by strongly increasing splenic Treg percentages, while slightly decreasing their function. Noteworthy, the mild negative effects of *M. vaccae* ATCC 15483^T^ on the capacity of Tregs isolated from SHC males to suppress proliferation of stimulated carboxyfluorescein succinimidyl ester-labeled responder cells (Fig. S3b) provides an explanation why "Treg suppressive capacity per animal" (Fig. 2k) is reduced in SHC male offspring of *M. vaccae* ATCC 15483^T^ vs. BBS dams. The latter again is in line with adjuvant-like effects of *M. vaccae* ATCC 15483^T^ on the immune system reported above and earlier.^2, 8, 23^

Noteworthy, as reported for CD4^+^CD25^+^FoxP3^+^ Tregs in percent of CD4^+^CD25^+^ splenocytes in CSC-exposed male offspring, SIP-exposed female offspring of *M. vaccae* ATCC 15483^T^ vs. BBS dams showed increased splenic CD4^+^CD25^+^FoxP3^+^ Tregs in percent of all splenocytes (Fig. S2a, Fig. S3m).

***M. vaccae* ATCC 15483^T^ intergenerationally diversifies male offspring’s fecal microbiome and increases the relative abundance of taxa known to produce SCFAs.**

As *M. vaccae* ATCC 15483^T^ intergenerationally also promoted the expansion of taxa (Fig. S4a) associated with pathology and/ or inflammation (i.e., Desulfobacterota; Desulfovibrionales/ Desulfovibrionacae)^24-27^ or decreased taxa referred to as beneficial gut commensals or probiotics (i.e., Bacteroidota/ Bacteroidales/ Bacteroidaceae & Muribaculaceae/*Muribaculum*; Lactobacillales/ Lactobacillaceae/ *Lactobacillus; Lachnospiraceae bacterium COE1*),^28-37^ it is likely that not only the immunoregulatory but also the adjuvant-like effects of *M. vaccae* ATCC 15483^T^ reported above are mediated by the gut microbiome.

As comparable effects were not detected after CSC, the intergenerational effects of *M. vaccae* ATCC 15483^T^ on bacterial taxa mediating the adjuvant-like effects of *M. vaccae* ATCC 15483^T^ seem to be diminished during CSC exposure.

***M. vaccae* ATCC 15483^T^ affects dams’ fecal microbiome**.

Fecal microbial evenness (i.e., Pielou's evenness), a metric for α-diversity (Fig. 4b-e), increased over time between mating and weaning in both the BBS and *M. vaccae* ATCC 15483^T^ group (Fig. 4e; significant main effect of time, Tab. S2), with the effect being more pronounced in the latter. Interestingly, metrics of gut microbiota α-diversity slightly increase over the course of gestation in sows, with an additional strong increase from pregnancy to weaning.^38^

Separate ANCOM-BC2 analysis of the fecal microbial composition at mating, birth and weaning revealed a lower relative abundance of Butyricoccaceae and Desulfovibrio in dams only at the latter time point (Fig. S5h). Noteworthy, while SCFA-producing taxa (i.e., Oscillospirales, Clostridia vadin BB60 group, *Roseburia,* Ruminococcaceae) and Desulfobacterota/ Desulfovibrionales/ Desulfovibrionaceae were both increased in their male offspring, *M. vaccae* ATCC 15483^T^ vs. BBS dams showed a lower relative abundance of the SCFA-producing taxon Butyricoccaceae^39-41^ and the taxon Desulfovibrio at weaning. Although we can only speculate about the underlying mechanisms, SCFA concentrations, which we hypothesize to be reduced in mothers that had received repeated administration of *M. vaccae* ATCC 15483^T^ vs. BBS and increased in their respective male offspring, might play a critical role. Given the coprophagic nature of mice,^42, 43^ together with the SCFAs acetate, propionate, and butyrate representing the most abundant microbiome-produced molecules in the gut,^44, 45^ fetal SCFAs levels are thought to depend on maternal fecal SCFA concentrations. Moreover, SCFA-supplemented fecal cultures displayed a reduction in the predicted abundance of SCFA producers, suggesting a possible undefined negative feedback mechanism.^46^ Although future studies are clearly warranted, we hypothesize that the SCFA concentrations that were fecal-orally transferred to the offspring from dams that had received repeated administration of *M. vaccae* ATCC 15483^T^ vs. BBS are lower, resulting in reduced systemic and intestinal SCFA levels in respective male offspring. The consequence would be a compromised negative feedback inhibition on the abundance of SCFA producers, facilitating the bloom of latter taxa.

***M. vaccae* ATCC 15483^T^ does not affect maternal behavior and physiology/immunology**

We did not reveal any differences between the groups in the number of delivered pups (Fig. S5a), body weight per litter at birth (Fig. S5b), maternal behavior (i.e., observation intervals with dam on nest: Fig. S5c; pup-retrieval test: latency to retrieve first pup, Fig. S5d; latency to pups back in nest, Fig. S5e), body weight per litter at weaning (Fig. S5f) and the number of weaned male and female pups (Fig. S5g). Note, the number of observational intervals spent on nest declined over time in both groups (i.e., main effect of time in *M. vaccae* ATCC 15483^T^ group, Tab. S2), which has been reported previously^3^ and is related to improved thermoregulation of the young and reduced frequency of required nursing intervals. Moreover, our data further did not indicate any differences in general (i.e., open-field/ novel object test) and social (i.e., social preference/ avoidance test) anxiety-related behavior, depressive-like behavior (i.e., saccharine preference test), adrenal, thymus and spleen weights, plasma corticosterone concentrations as well as in *ex vivo* splenocyte cell viability, LPS responsiveness and GC resistance between *M. vaccae* ATCC 15483^T^- and BBS-administered dams after weaning of their pups (data not shown in the current manuscript). Finally, C3 mRNA expression in breast tissue of BBS- and *M. vaccae* ATCC 15483^T^-administered dams was comparable between the groups (Fig. S6a, b).

**2. Supplementary Tables**

**Supplementary Table S1:**

Detailed description of animal numbers per group. Abbreviations: borate-buffered saline (BBS), *Mycobacterium (M.) vaccae* ATCC 15483^T^ (MvacATCC), single-housed control (SHC), chronic subordinate colony housing (CSC), group-housed control (GHC), social instability paradigm (SIP).

|  | Groups | | | |
| --- | --- | --- | --- | --- |
| **Experiment 1** | **BBS** | | **MvacATCC** | |
| dams | 10 | | 10 | |
| **Experiment 1** | **BBS** | | **MvacATCC** | |
| male offspring | **SHC**  12 | **CSC**  12 | **SHC**  9 | **CSC**  12 |
| female offspring | **GHC**  12 | **SIP**  12 | **GHC**  8 | **SIP**  10 |
| **Experiment 2** | **BBS** | | **MvacATCC** | |
| dams | 5 | | 7 | |

**Supplementary Table S2:**

Detailed description of all statistical analysis and effects shown in Figures 2-3 and Figures S1, S3-S7.

| Fig. 2:  **Experiment 1**  **Physiology and**  **Immunology**  in males | a) Relative adrenal weight | 2WayANOVA; no outlier |
| --- | --- | --- |
|  |  | F(Chronic adult stress)_1,41_ =83.66;  *P* < 0.001 |
|  |  | F(Treatment)_1,41_ = 9.779; *P* = 0.003 |
|  |  | F(Chronic adult stress*Treatment)_1,41_ = 6.652;  *P* = 0.014 |
|  |  | BBS: SHC vs. CSC *P* < 0.001 *** |
|  |  | MvacATCC: SHC vs. CSC  *P* < 0.001 *** |
|  |  | CSC: BBS vs. MvacATCC  *P* < 0.001 *** |
|  | b) Relative spleen weight | MWU |
|  |  | BBS: SHC vs. CSC *P* < 0.001 *** |
|  |  | MvacATCC: SHC vs. CSC  *P* < 0.001 *** |
|  |  | CSC: BBS vs. MvacATCC  *P* = 0.033 * |
|  | c) Relative thymus weight | 2WayANOVA; no outlier |
|  |  | F(Chronic adult stress)_1,41_ =11.59;  *P* = 0.001 |
|  |  | F(Treatment)_1,41_ = 19.78; *P* < 0.001 |
|  |  | F(Chronic adult stress*Treatment)_1,41_  = 13.19; *P* < 0.001 |
|  |  | BBS: SHC vs. CSC *P* < 0.001 *** |
|  |  | CSC: BBS vs. MvacATCC  *P* < 0.001 *** |
|  | d) Tibia length | MWU |
|  |  | BBS: SHC vs. CSC *P* = 0.010 ** |
|  |  | CSC: BBS vs. MvacATCC  *P* = 0.005 ** |
|  | e) TLR2 concentration  in isolated splenocytes  [grey density] | 2WayANOVA; 1 outlier  (MvacATCC SHC) |
|  |  | F(Chronic adult stress)_1,40_ = 7.449;  *P* = 0.009 |
|  |  | BBS: SHC vs. CSC *P* = 0.016 * |
|  | f) TLR4 concentration  in isolated splenocytes  [grey density] | MWU |
|  |  | BBS: SHC vs. CSC *P* = 0.001 *** |
|  |  | CSC: BBS vs. MvacATCC  *P* = 0.024 * |
|  | g) Cell viability,  CORT 0 µM | MWU |
|  |  | Basal BBS: SHC vs. CSC  *P* = 0.001 *** |
|  |  | Basal CSC: BBS vs. MvacATCC  *P* = 0.001 *** |
|  |  | LPS BBS: SHC vs. CSC  *P* < 0.001 *** |
|  |  | LPS SHC: BBS vs. MvacATCC  *P* = 0.001 *** |
|  |  | Wilcoxon test |
|  |  | BBS SHC: basal vs. LPS  *P* < 0.001 *** |
|  |  | BBS CSC: basal vs. LPS  *P* < 0.001*** |
|  |  | MvacATCC SHC: basal vs. LPS  *P* = 0.004 ** |
|  |  | MvacATCC CSC: basal vs. LPS  *P* = 0.002 ** |
|  | h) Delta cell viability  (LPS-basal),  CORT 0 µM | MWU |
|  |  | BBS SHC vs. CSC *P* < 0.001 *** |
|  |  | SHC BBS vs. MvacATCC *P* = 0.006 ** |
|  | i) Delta cell viability in %   of CORT,  CORT 0 µM set to 100% | Friedman test |
|  |  | BBS SHC: X^2^ (2, 11) = 11.09,  *P* = 0.003  Dunn’s: CORT 0.1µM *P* = 0.017 *  CORT 5.0µM *P* = 0.009 ** |
|  |  | BBS CSC: X^2^ (2, 12) = 16.67,  *P* < 0.001  Dunn’s: CORT 5.0µM  *P* < 0.001 *** |
|  |  | MvacATCC SHC: X^2^ (2, 9) = 14.00,  *P* < 0.001  Dunn’s: CORT 0.1µM *P* = 0.014 *  CORT 5.0µM *P* = 0.001 *** |
|  |  | MvacATCC CSC:  X^2^ (2, 10) = 15.20, *P* < 0.001  Dunn’s: CORT 0.1µM  *P* < 0.001 ***  CORT 5.0µM *P* = 0.005 ** |
|  |  | MWU in CORT 0.1µM |
|  |  | SHC BBS vs. MvacATCC  *P* = 0.046 * |
|  |  | CSC BBS vs. MvacATCC  *P* = 0.003 ** |
|  |  | MWU in CORT 5.0µM |
|  |  | CSC BBS vs. MvacATCC  *P* = 0.021 * |
|  | j) CD4^+^CD25^+^FoxP3^+^ Tregs  [in % of all cells] | 2WayANOVA; no outlier |
|  |  | F(Chronic adult stress)_1,41_ = 15.58;  *P* < 0.001 |
|  |  | F(Treatment)_1,41_ = 4.627; *P* = 0.037 |
|  |  | F(Interaction)_1,41_ = 10.58; *P* = 0.002 |
|  |  | BBS: SHC vs. CSC *P* < 0.001*** |
|  |  | CSC: BBS vs. MvacATCC  *P* < 0.001 *** |
|  | k) Treg suppression capacity per animal | 2WayANOVA; no outlier |
|  |  | F(Interaction)_1,41_= 13.55; *P* = 0.001 |
|  |  | F(Stress)_1,41_= 34.36; *P* = 0.001 |
|  |  | BBS: SHC vs. CSC *P* = 0.001 *** |
|  |  | SHC: BBS vs. MvacATCC  *P* = 0.024 * |
|  |  | CSC: BBS vs. MvacATCC  *P* = 0.027 * |
|  | l) Chemotactic activity  of isolated WBCs | MWU |
|  |  | BBS: SHC vs. CSC *P* = 0.002 ** |
|  |  | MvacATCC: SHC vs. CSC  *P* = 0.002 ** |
|  |  | CSC: BBS vs. MvacATCC  *P* = 0.002 ** |
|  | m) Monocytes/MO-MDSCs  [in % of counted cells] | MWU |
|  |  | BBS: SHC vs. CSC *P* < 0.001 *** |
|  |  | MvacATCC: SHC vs. CSC  *P* = 0.001 *** |
|  |  | SHC: BBS vs. MvacATCC  *P* = 0.043 * |
|  | n) Neutrophils  [in % of counted cells] | MWU |
|  |  | BBS: SHC vs. CSC *P* = 0.007 ** |
|  |  | MvacATCC: SHC vs. CSC  *P* = 0.060 (*) |
|  | o) PMN-MDSCs  [in % of counted cells] | MWU |
|  |  | BBS: SHC vs. CSC *P* < 0.001*** |
|  |  | MvacATCC: SHC vs. CSC  *P* < 0.001 *** |
|  | | |
| Fig. 3:  **Experiment 1**  **Fecal microbiome analysis** prior to and after CSC  in males | a) Beta diversity   prior to CSC | PERMANOVA – Bray Curtis |
|  |  | Pseudo-*F* = 6.4497; *P* = 0.001 *** |
|  | b) Observed ASVs   prior to CSC | Unpaired t-test with Welch’s correction;  no outlier |
|  |  | BBS vs. MvacATCC: *P* < 0.001 *** |
|  | c) Shannon diversity index  prior to CSC | Unpaired t-test; no outlier |
|  |  | BBS vs. MvacATCC: *P* = 0.001 *** |
|  | d) Chao1 index   prior to CSC | Unpaired t-test; no outlier |
|  |  | BBS vs. MvacATCC: *P* < 0.001 *** |
|  | e) Pielou’s evenness   prior to CSC | Unpaired t-test; no outlier |
|  |  | n. s. |
|  | f) Correlation analysis – Alpha diversity metrics (observed ASVs, Chao1, Shannon) prior to CSC with physiological and immunological parameters in BBS and MvacATCC groups | Spearman |
|  |  | for detailed rho- and *P*-values see Fig. 3f |
|  | g) Beta diversity  after CSC | PERMANOVA – Bray Curtis |
|  |  | Pseudo-*F* = 2.5185; *P* = 0.003 ** |
|  | h) Observed ASVs  after CSC | Unpaired *t*-test; no outlier |
|  |  | BBS vs. MvacATCC: *P* < 0.001 *** |
|  | i) Shannon diversity index  after CSC | Unpaired t-test; no outlier |
|  |  | BBS vs. MvacATCC: *P* = 0.045 * |
|  | j) Chao1 index  after CSC | Unpaired t-test; no outlier |
|  |  | BBS vs. MvacATCC: *P* < 0.001 *** |
|  | k) Pielou’s evenness  after CSC | Unpaired *t*-test; no outlier |
|  |  | n. s. |
|  | l) Correlation analysis – Alpha diversity metrics (observed ASVs, Chao1, Shannon) after CSC with physiological and immunological parameters in BBS and MvacATCC groups | Spearman |
|  |  | for detailed rho- and *P*-values see Fig. 3l |
|  | | |
| Fig. 4  **Experiment 1**  **Maternal fecal microbiome analysis** at mating, birth and weaning | a) Beta diversity | PERMANOVA – Bray Curtis |
|  |  | Mating: Pseudo-*F* = 2.4099;  *P* = 0.016 * |
|  |  | Birth: Pseudo-*F* = 1.4053; *P* = 0.145 |
|  |  | Weaning: Pseudo-*F* = 1.8161;  *P* = 0.010 ** |
|  | b) Observed ASVs | Friedman test |
|  |  | n. s. |
|  |  | MWU – Weaning |
|  |  | BBS vs. MvacATCC *P* = 0.038 * |
|  | c) Shannon | Friedman test |
|  |  | n. s. |
|  |  | MWU |
|  |  | n. s. |
|  | d) Chao1 | Friedman test |
|  |  | n. s. |
|  |  | MWU - Weaning |
|  |  | BBS vs. MvacATCC *P* = 0.036 * |
|  | e) Pielou’s evenness | LMM; no outlier |
|  |  | *F*(Time)_1.507, 22.61_ = 3.903; *P* = 0.045 |
|  |  | MvacATCC:  Weaning vs. Birth *P* = 0.018 * |
|  | | |
| Fig. S1:  **Experiment 1**  **Behaviors**  in males and females | a) Social preference  in males | MWU |
|  |  | n. s. |
|  | b) Social preference  in females | MWU |
|  |  | n. s. |
|  | c) OF – Distance moved  in males | MWU |
|  |  | n. s. |
|  | d) OF – Entries to inner zone in males | 2WayANOVA; no outlier |
|  |  | n. s. |
|  |  | Effect size:  partial η^2^ (Interaction) = 0.00248  partial η^2^ (Treatment) = 0.00085  partial η^2^ (Stress) = 0.00095 |
|  | e) OF – Time in inner zone in males | 2WayANOVA; 1 outlier (MvacATCC CSC) |
|  |  | n. s. |
|  |  | Effect size:  partial η^2^ (Interaction) = 0.89495  partial η^2^ (Treatment) = 0.01148  partial η^2^ (Stress) = 0.02959 |
|  | f) NO – Entries into contact zone in males | MWU |
|  |  | BBS: SHC vs. CSC *P* = 0.034 * |
|  |  | CSC: BBS vs. MvacATCC  *P* = 0.002 ** |
|  | g) NO – Time in contact zone in males | MWU |
|  |  | CSC: BBS vs. MvacATCC  *P* < 0.001 *** |
|  | h) OF – Distance moved  in females | MWU |
|  |  | BBS: GHC vs. SIP *P* = 0.045 * |
|  | i) OF – Entries to inner zone in females | 2WayANOVA; no outlier |
|  |  | *F*(Interaction)_1,37_ = 3.487, *P* = 0.070 |
|  |  | Effect size:  partial η^2^ (Interaction) = 0.01273  partial η^2^ (Treatment) = 0.01052  partial η^2^ (Stress) = 0.00018 |
|  | j) OF – Time in contact zone in females | MWU |
|  |  | MvacATCC: GHC vs. SIP  *P* = 0.055 (*) |
|  | k) NO – Entries to contact zone in females | 2WayANOVA; 1 outlier (MvacATCC GHC) |
|  |  | n. s. |
|  |  | Effect size:  partial η^2^ (Interaction) = 0.17518  partial η^2^ (Treatment) = 0.00680  partial η^2^ (Stress) = 0.01663 |
|  | l) NO – Time in contact zone in females | MWU |
|  |  | n. s. |
|  | | |
| Fig. S3:  **Experiment 1**  **Physiology and Immunology**  in males and females | a) 48 h – Proliferating cells [% of stained cells with basal set to 100%]  in males | Wilcoxon test |
|  |  | basal vs. aCD3/28 *P* = 0.031 * |
|  | b) 48 h – Proliferating cells [% of stained cells with aCD3/28 set to 100%]  in males | Wilcoxon test |
|  |  | BBS: SHC vs. aCD3/28 *P* = 0.031 * |
|  |  | BBS: CSC vs. aCD3/28 *P* = 0.031 * |
|  |  | MvacATCC: SHC vs. aCD3/28  *P* = 0.031 * |
|  |  | MWU |
|  |  | SHC: BBS vs. MvacATCC  *P* = 0.004 ** |
|  |  | CSC: BBS vs. MvacATCC  *P* = 0.002 ** |
|  | c) CD4^+^CD25^+^FoxP3^+^ Tregs  [in % of CD4^+^CD25^+^  cells] in males | 2WayANOVA; no outlier |
|  |  | F(Treatment)_1,41_= 8.068; *P* = 0.007 |
|  |  | CSC: BBS vs. MvacATCC  *P* = 0.014 * |
|  | d) Relative adrenal weight  in females | 2WayANOVA; no outlier |
|  |  | n. s. |
|  | e) Relative spleen weight  in females | MWU |
|  |  | n. s. |
|  | f) Relative thymus weight  in females | 2WayANOVA; no outlier |
|  |  | n. s. |
|  | g) Tibia length in females | MWU |
|  |  | n. s. |
|  | h) TLR2 concentration  in isolated splenocytes  [grey density] in females | MWU |
|  |  | n. s. |
|  | i) TLR4 concentration  in isolated splenocytes  [grey density] in females | MWU |
|  |  | n. s. |
|  | j) Cell viability,  CORT 0 µM in females | MWU |
|  |  | n. s. |
|  |  | Wilcoxon test |
|  |  | BBS GHC: basal vs. LPS  *P* < 0.001 *** |
|  |  | BBS SIP: basal vs. LPS  *P* < 0.001 *** |
|  |  | MvacATCC GHC: basal vs. LPS  *P* = 0.008 ** |
|  |  | MvacATCC SIP: basal vs. LPS  *P* = 0.002 ** |
|  | k) Delta cell viability  (LPS-basal),  CORT 0 µM in females | 2WayANOVA; 1 outlier (BBS SIP) |
|  |  | F(Treatment)_1,37_ = 4.086; *P* = 0.051 |
|  | l) Delta cell viability  (LPS-basal),  CORT 0 µM in females | Friedman test |
|  |  | BBS GHC: X^2^ (2, 11) = 16.91,  *P* < 0.001  Dunn’s: CORT 0.1µM *P* = 0.004 **  CORT 5.0µM  *P* < 0.001 *** |
|  |  | BBS SIP: X^2^ (2, 12) = 18.67,  *P* < 0.001  Dunn’s: CORT 0.1µM *P* = 0.003 **  CORT 5.0µM  *P* < 0.001 *** |
|  |  | MvacATCC GHC:  X^2^ (2, 8) = 10.90, *P* = 0.002  Dunn’s: CORT 0.1µM *P* = 0.018 *  CORT 5.0µM *P* = 0.012 * |
|  |  | MvacATCC SIP: X^2^ (2, 9) = 16.22,  *P* < 0.001  Dunn’s: CORT 0.1µM *P* = 0.055 (*)  CORT 5.0µM  *P* < 0.001 *** |
|  |  | MWU in CORT 0.1µM |
|  |  | n. s. |
|  |  | MWU in CORT 5.0µM |
|  |  | n. s. |
|  | m) CD4^+^CD25^+^FoxP3^+^  Tregs  [in % of counted cells]  in females | 2WayANOVA; no outlier |
|  |  | F(Treatment)_1,38_= 7.455; *P* = 0.010 |
|  |  | SIP: BBS vs. MvacATCC  *P* = 0.029 * |
|  | n) CD4^+^CD25^+^FoxP3^+^  Tregs [in % of  CD4^+^CD25^+^ cells]  in females | 2WayANOVA; no outlier |
|  |  | n. s. |
|  | o) 48 h – Proliferating cells [% of stained cells with basal set to 100%]  in females | Wilcoxon test |
|  |  | basal vs. aCD3/28 *P* = 0.031 * |
|  | p) 48 h – Proliferating cells  [% of stained cells with  aCD3/28 set to 100%]  in females | Wilcoxon test |
|  |  | n. s. |
|  |  | MWU |
|  |  | n. s. |
|  | q) CD4^+^CD25^+^FoxP3^+^ Treg  suppression capacity  per animal in females | MWU |
|  |  | n. s. |
|  | r) Neutrophil chemotactic  activity in females | 2WayANOVA; no outlier |
|  |  | n. s. |
|  | s) Neutrophils  [in % of counted cells]  in females | 2WayANOVA; 1 outlier  (MvacATCC SIP) |
|  |  | n. s. |
|  | t) PMN-MDSCs  [in % of counted cells]  in females | MWU |
|  |  | n. s. |
|  | u) Monocytes/ MO-MDSCs  [in % of counted cells]  in females | 2WayANOVA; 3 outliers (BBS GHC, MvacATCC GHC, MvacATCC SIP) |
|  |  | F(Chronic adult stress)_1,35_ = 14.76;  *P* < 0.001 |
|  |  | BBS: GHC vs. SIP *P* = 0.010 ** |
|  |  | MvacATCC: GHC vs. SIP  *P* = 0.034 * |
|  | | |
| Fig. S4:  **Experiment 1**  **Community composition** analysis prior to and after CSC in males | a) Bacterial taxa (above 0.1% rel. abundance) prior to CSC sorted for: | |
|  | **Phyla** | |
|  | – Bacteroidota | ANCOM-BC:  LFC_groupMvacATCC: -0.703  *P* (FDR corrected) = 0.002 ** |
|  | – Desulfobacterota | ANCOM-BC:  LFC_groupMvacATCC: 1.712  *P* (FDR corrected) < 0.001 *** |
|  | **Orders** | |
|  | – Bacteroidales | ANCOM-BC:  LFC_groupMvacATCC: -0.703  *P* (FDR corrected) = 0.007 ** |
|  | – Lactobacillales | ANCOM-BC:  LFC_groupMvacATCC: -1.825  *P* (FDR corrected) = 0.012 * |
|  | – Oscillospirales | ANCOM-BC:  LFC_groupMvacATCC: 1.808  *P* (FDR corrected) < 0.001 *** |
|  | – Clostridia vadin  BB60 group | ANCOM-BC:  LFC_groupMvacATCC: 1.272  *P* (FDR corrected) = 0.010 ** |
|  | – Desulfovibrionales | ANCOM-BC:  LFC_groupMvacATCC: 1.712  *P* (FDR corrected) = 0.001 *** |
|  | **Families** | |
|  | – Bacteroidaceae | ANCOM-BC:  LFC_groupMvacATCC: -0.955  *P* (FDR corrected) = 0.048 * |
|  | – Lactobacillaceae | ANCOM-BC:  LFC_groupMvacATCC: -1.825  *P* (FDR corrected) = 0.020 * |
|  | – Muribaculaceae | ANCOM-BC:  LFC_groupMvacATCC: -0.789  *P* (FDR corrected) = 0.010 ** |
|  | – Desulfovibrionaceae | ANCOM-BC:  LFC_groupMvacATCC: 1.712  *P* (FDR corrected) = 0.003 ** |
|  | **Genera** | |
|  | – *Lactobacillus* | ANCOM-BC:  LFC_groupMvacATCC: -2.239  *P* (FDR corrected) = 0.008 ** |
|  | – *Muribaculum* | ANCOM-BC:  LFC_groupMvacATCC: -0.897  *P* (FDR corrected) = 0.050 * |
|  | – *Roseburia* | ANCOM-BC:  LFC_groupMvacATCC: 1.480  *P* (FDR corrected) = 0.036 * |
|  | **Species** | |
|  | – *Lachnospiraceae_bacterium*  *_COE1* | ANCOM-BC:  LFC_groupMvacATCC: -1.761  *P* (FDR corrected) = 0.019 * |
|  | b) Correlation analysis – FoxP3^+^ Tregs (% of all cells) and Treg suppression capacity per animal with respective phyla, order, family, genera and species, (known to be SCFA-producers) in BBS and MvacATCC groups prior to CSC | Spearman |
|  |  | for detailed rho- and *P*-values  see Fig. S4b |
|  | c) Bacterial taxa (above 0.1% rel. abundance) after CSC sorted for: | |
|  | **Families** | |
|  | – Ruminococcaceae | ANCOM-BC:  LFC_groupMvacATCC: 1.275  *P* (FDR corrected) = 0.008 ** |
|  | d) Correlation analysis – FoxP3^+^ Tregs (% of all cells) and Treg suppression capacity per animal with respective families (known to be SCFA-producers) in BBS and MvacATCC groups after CSC | Spearman |
|  |  | for detailed rho- and *P*-values  see Fig. S4d |
|  | | |
| Fig. S5:  **Experiment 1**  **Maternal behavior and pups** | a) Numbers of pups at birth | MWU |
|  |  | n. s. |
|  | b) Body weight per litter  at birth | MWU |
|  |  | n. s. |
|  | c) Observation intervals  spent on nest, total  observation  intervals set to 100% | Friedman test |
|  |  | BBS: X^2^ (4, 10) = 24.91, *P* < 0.001  Dunn’s: LD1 vs. LD4 *P* = 0.007 **  LD1 vs. LD5 *P* = 0.002 **  LD1 vs. LD6 *P* = 0.001 *** |
|  |  | MvacATCC: X^2^ (4, 8) = 12.03, *P* = 0.017  Dunn’s: n. s. |
|  |  | MWU |
|  |  | n. s. |
|  | d) Latency to retrieve  1^st^ pup | MWU |
|  |  | n. s. |
|  | e) Latency to pups back  in nest | Friedman test |
|  |  | n. s. |
|  |  | MWU |
|  |  | n. s. |
|  | f) Body weight per litter  at weaning | MWU |
|  |  | n. s. |
|  | g) Numbers of male and  female pups at weaning | 2WayANOVA; no outlier |
|  |  | n. s. |
|  | h) Bacterial taxa (above 0.1% rel. abundance) at weaning sorted for: | |
|  | **Family** | |
|  | – Butyricoccaceae | ANCOM-BC:  LFC_groupMvacATCC: -1.766  *P* (FDR corrected) = 0.038 * |
|  | **Genus** | |
|  | – *Desulfovibrio* | ANCOM-BC:  LFC_groupMvacATCC: -2.353  *P* (FDR corrected) = 0.043 * |
|  | | |
| Fig. S6  **Experiment 2**  **Maternal complement C3** expression in mammary glands | a) Graphical timeline of dams (Experiment 2) | |
|  | b) Relative complement C3  expression  in mammary glands | MWU |
|  |  | n. s. |
|  | | |

Supplementary References

1.	Schiele J, Tsai PL, Ernst G, Blank C, Beck S, Schimmele T *et al.* Mycobacterium vaccae ATCC 15483(T) administered to nulliparous C57BL/6N mice prior to mating protects their male offspring against the negative consequences of prenatal stress. *Brain Behav Immun* 2025; **131:** 106169.

2.	Langgartner D, Amoroso M, Kempter E, Kustermann M, Scheurer J, Lowry CA *et al.* Mycobacterium vaccae protects against glucocorticoid resistance resulting from combined physical and psychosocial trauma in mice. *Brain Behav Immun* 2023; **109:** 221-234.

3.	Hillerer KM, Reber SO, Neumann ID, Slattery DA. Exposure to chronic pregnancy stress reverses peripartum-associated adaptations: implications for postpartum anxiety and mood disorders. *Endocrinology* 2011; **152**(10)**:** 3930-3940.

4.	Hillerer KM, Neumann ID, Slattery DA. From stress to postpartum mood and anxiety disorders: how chronic peripartum stress can impair maternal adaptations. *Neuroendocrinology* 2012; **95**(1)**:** 22-38.

5.	Pardon M, Gérardin P, Joubert C, Pérez-Diaz F, Cohen-Salmon C. Influence of prepartum chronic ultramild stress on maternal pup care behavior in mice. *Biol Psychiatry* 2000; **47**(10)**:** 858-863.

6.	Booher WC, Hall LA, Thomas AL, Merhroff EA, Reyes Martínez GJ, Scanlon KE *et al.* Anxiety-related defensive behavioral responses in mice selectively bred for High and Low Activity. *Genes Brain Behav* 2021; **20**(7)**:** e12730.

7.	Langgartner D, Peterlik D, Foertsch S, Füchsl AM, Brokmann P, Flor PJ *et al.* Individual differences in stress vulnerability: The role of gut pathobionts in stress-induced colitis. *Brain Behav Immun* 2017; **64:** 23-32.

8.	Mazzari G, Lowry CA, Langgartner D, Reber SO. Subcutaneous Mycobacterium vaccae ameliorates the effects of early life adversity alone or in combination with chronic stress during adulthood in male and female mice. *Neurobiol Stress* 2023; **26:** 100568.

9.	Amoroso M, Bottcher A, Lowry CA, Langgartner D, Reber SO. Subcutaneous Mycobacterium vaccae promotes resilience in a mouse model of chronic psychosocial stress when administered prior to or during psychosocial stress. *Brain Behav Immun* 2020; **87:** 309-317.

10.	Slattery DA, Uschold N, Magoni M, Bär J, Popoli M, Neumann ID *et al.* Behavioural consequences of two chronic psychosocial stress paradigms: anxiety without depression. *Psychoneuroendocrinology* 2012; **37**(5)**:** 702-714.

11.	Langgartner D, Koenen M, Kupfer S, Glogger L, Kurz L, Perez-Rivas LG *et al.* Intact GR dimerization is critical for restraining plasma ACTH levels during chronic psychosocial stress. *Neurobiol Stress* 2023; **24:** 100541.

12.	Heinrichs SC, Koob GF. Application of experimental stressors in laboratory rodents. *Curr Protoc Neurosci* 2006; **Chapter 8:** Unit8.4.

13.	Singewald GM, Nguyen NK, Neumann ID, Singewald N, Reber SO. Effect of chronic psychosocial stress-induced by subordinate colony (CSC) housing on brain neuronal activity patterns in mice. *Stress* 2009; **12**(1)**:** 58-69.

14.	Kappel S, Hawkins P, Mendl MT. To Group or Not to Group? Good Practice for Housing Male Laboratory Mice. *Animals (Basel)* 2017; **7**(12).

15.	Schiele J, Mazzari G, Struck A, Bailer Y, Langgartner D, Reber SO. Chronic sensory contact with subordinated conspecifics promotes splenic glucocorticoid resistance in experimentally wounded C57BL/6N male mice. *Sci Rep* 2024; **14**(1)**:** 10867.

16.	Langgartner D, Füchsl AM, Kaiser LM, Meier T, Foertsch S, Buske C *et al.* Biomarkers for classification and class prediction of stress in a murine model of chronic subordination stress. *PLoS One* 2018; **13**(9)**:** e0202471.

17.	Collison LW, Vignali DA. In vitro Treg suppression assays. *Methods Mol Biol* 2011; **707:** 21-37.

18.	Li Y, Horst K, Greven J, Mert Ü, Lupu L, Palmer A *et al.* Modulation of the hepatic RANK-RANKL-OPG axis by combined C5 and CD14 inhibition in a long-term polytrauma model. *Front Immunol* 2024; **15:** 1434274.

19.	Schmittgen TD, Livak KJ. Analyzing real-time PCR data by the comparative C(T) method. *Nat Protoc* 2008; **3**(6)**:** 1101-1108.

20.	Grubbs FE. Procedures for Detecting Outlying Observations in Samples. *Technometrics* 1969; **11**(1)**:** 1-21.

21.	Foertsch S, Haffner-Luntzer M, Kroner J, Gross F, Kaiser K, Erber M *et al.* Chronic psychosocial stress disturbs long-bone growth in adolescent mice. *Dis Model Mech* 2017; **10**(12)**:** 1399-1409.

22.	Yohn CN, Ashamalla SA, Bokka L, Gergues MM, Garino A, Samuels BA. Social instability is an effective chronic stress paradigm for both male and female mice. *Neuropharmacology* 2019; **160:** 107780.

23.	Reber SO, Siebler PH, Donner NC, Morton JT, Smith DG, Kopelman JM *et al.* Immunization with a heat-killed preparation of the environmental bacterium Mycobacterium vaccae promotes stress resilience in mice. *Proc Natl Acad Sci U S A* 2016; **113**(22)**:** E3130-3139.

24.	Voigt RM, Zalta AK, Raeisi S, Zhang L, Brown JM, Forsyth CB *et al.* Abnormal intestinal milieu in posttraumatic stress disorder is not impacted by treatment that improves symptoms. *American Journal of Physiology-Gastrointestinal and Liver Physiology* 2022; **323**(2)**:** G61-G70.

25.	Figliuolo VR, dos Santos LM, Abalo A, Nanini H, Santos A, Brittes NM *et al.* Sulfate-reducing bacteria stimulate gut immune responses and contribute to inflammation in experimental colitis. *Life Sci* 2017; **189:** 29-38.

26.	Tamargo A, Molinero N, Reinosa JJ, Alcolea-Rodriguez V, Portela R, Bañares MA *et al.* PET microplastics affect human gut microbiota communities during simulated gastrointestinal digestion, first evidence of plausible polymer biodegradation during human digestion. *Scientific Reports* 2022; **12**(1)**:** 528.

27.	Rajput M, Momin T, Singh A, Banerjee S, Villasenor A, Sheldon J *et al.* Determining the association between gut microbiota and its metabolites with higher intestinal Immunoglobulin A response. *Veterinary and Animal Science* 2023; **19:** 100279.

28.	Han B, Shi L, Bao MY, Yu FL, Zhang Y, Lu XY *et al.* Dietary ellagic acid therapy for CNS autoimmunity: Targeting on Alloprevotella rava and propionate metabolism. *Microbiome* 2024; **12**(1)**:** 114.

29.	Wang Y, Guo Y, Chen H, Wei H, Wan C. Potential of Lactobacillus plantarum ZDY2013 and Bifidobacterium bifidum WBIN03 in relieving colitis by gut microbiota, immune, and anti-oxidative stress. *Can J Microbiol* 2018; **64**(5)**:** 327-337.

30.	Tofani GSS, Leigh S-J, Gheorghe CE, Bastiaanssen TFS, Wilmes L, Sen P *et al.* Gut microbiota regulates stress responsivity via the circadian system. *Cell Metab* 2024.

31.	Gryaznova M, Dvoretskaya Y, Burakova I, Syromyatnikov M, Popov E, Kokina A *et al.* Dynamics of Changes in the Gut Microbiota of Healthy Mice Fed with Lactic Acid Bacteria and Bifidobacteria. *Microorganisms* 2022; **10**(5)**:** 1020.

32.	Wexler HM. <i>Bacteroides</i>: the Good, the Bad, and the Nitty-Gritty. *Clin Microbiol Rev* 2007; **20**(4)**:** 593-621.

33.	Round JL, Mazmanian SK. The gut microbiota shapes intestinal immune responses during health and disease. *Nat Rev Immunol* 2009; **9**(5)**:** 313-323.

34.	Hill C, Guarner F, Reid G, Gibson GR, Merenstein DJ, Pot B *et al.* The International Scientific Association for Probiotics and Prebiotics consensus statement on the scope and appropriate use of the term probiotic. *Nature Reviews Gastroenterology & Hepatology* 2014; **11**(8)**:** 506-514.

35.	Karimi K, Inman MD, Bienenstock J, Forsythe P. Lactobacillus reuteri-induced regulatory T cells protect against an allergic airway response in mice. *Am J Respir Crit Care Med* 2009; **179**(3)**:** 186-193.

36.	Savino F, Galliano I, Garro M, Savino A, Daprà V, Montanari P *et al.* Regulatory T cells and Toll-like receptor 2 and 4 mRNA expression in infants with colic treated with Lactobacillus reuteri DSM17938. *Benef Microbes* 2018; **9**(6)**:** 917-925.

37.	Savino F, Garro M, Montanari P, Galliano I, Bergallo M. Crying Time and RORγ/FOXP3 Expression in Lactobacillus reuteri DSM17938-Treated Infants with Colic: A Randomized Trial. *J Pediatr* 2018; **192:** 171-177.e171.

38.	Ji YJ, Li H, Xie PF, Li ZH, Li HW, Yin YL *et al.* Stages of pregnancy and weaning influence the gut microbiota diversity and function in sows. *J Appl Microbiol* 2019; **127**(3)**:** 867-879.

39.	Schwerdtfeger LA, Montini F, Lanser TB, Ekwudo MN, Zurawski J, Tauhid S *et al.* Gut microbiota and metabolites are linked to disease progression in multiple sclerosis. *Cell Rep Med* 2025; **6**(4)**:** 102055.

40.	Chang SC, Shen MH, Liu CY, Pu CM, Hu JM, Huang CJ. A gut butyrate-producing bacterium Butyricicoccus pullicaecorum regulates short-chain fatty acid transporter and receptor to reduce the progression of 1,2-dimethylhydrazine-associated colorectal cancer. *Oncol Lett* 2020; **20**(6)**:** 327.

41.	Eeckhaut V, Machiels K, Perrier C, Romero C, Maes S, Flahou B *et al.* Butyricicoccus pullicaecorum in inflammatory bowel disease. *Gut* 2013; **62**(12)**:** 1745-1752.

42.	Morais LH, Golubeva AV, Moloney GM, Moya-Pérez A, Ventura-Silva AP, Arboleya S *et al.* Enduring Behavioral Effects Induced by Birth by Caesarean Section in the Mouse. *Curr Biol* 2020; **30**(19)**:** 3761-3774.e3766.

43.	Robertson SJ, Lemire P, Maughan H, Goethel A, Turpin W, Bedrani L *et al.* Comparison of Co-housing and Littermate Methods for Microbiota Standardization in Mouse Models. *Cell Rep* 2019; **27**(6)**:** 1910-1919.e1912.

44.	Gregory AL, Pensinger DA, Hryckowian AJ. A short chain fatty acid-centric view of Clostridioides difficile pathogenesis. *PLoS Pathog* 2021; **17**(10)**:** e1009959.

45.	Macfarlane S, Macfarlane GT. Regulation of short-chain fatty acid production. *Proc Nutr Soc* 2003; **62**(1)**:** 67-72.

46.	Peterson CT, Perez Santiago J, Iablokov SN, Chopra D, Rodionov DA, Peterson SN. Short-Chain Fatty Acids Modulate Healthy Gut Microbiota Composition and Functional Potential. *Curr Microbiol* 2022; **79**(5)**:** 128.

2 Supplementary Figures and Supplementary Figure Legends

**Fig. S1 | Intergenerational effects of repeated i.g. administration of *Mycobacterium vaccae* ATCC 15483^T^ to female nulliparous C57BL/6N mice prior to mating on social- and general anxiety-related behavior of male and female offspring prior to and after exposure to chronic psychosocial stressor during adulthood (Experiment 1).**

The social preference/ avoidance test was performed prior to chronic adult stressor exposure in experimental **(a)** male and **(b)** female offspring. **a, b)** Social preference (time spent in the direct contact zone (during social cage exposure) divided by total time spent in direct contact zone (during empty + social cage exposure)). The open-field (OF)/ novel object (NO) test was performed at the end of chronic adult stress exposure in experimental male **(c-g)** and female **(h-l)** offspring. **c, h)** Distance moved during OF exposure. **d, i)** Entries into the inner zone during OF exposure. **e, j)** Time in the inner zone during OF exposure. **f, k)** Entries into contact zone during NO exposure. **g, l)** Time in the contact zone during NO exposure. Data are presented as mean + SEM including individual values (dots). Blue bars: male offspring (light blue: single-housed controls (SHC), dark blue: chronic subordinate colony housing (CSC)), red bars: female offspring (light red: group-housed controls (GHC); dark red: social instability paradigm (SIP)). Non-hatched bars: borate-buffered saline (BBS), hatched bars: *M. vaccae* ATCC 15483^T^ (MvacATCC); * *P* ≤ 0.05, ** *P* ≤ 0.01, *** *P* ≤ 0.001 vs. respective control condition. Social preference/ avoidance test: *N* = 15 – 24 per group; open-field/ novel object test: *N* = 7 – 12 per group (Tab. S1). In case individual samples had to be excluded due to methodological reasons, this is reported in the supplementary materials and methods (Sections 1.4). Exact *P* values and statistical tests employed are provided in Tab. S2.

**Fig. S2 | Regulatory T cells (Tregs) fluorescence-activated cell sorting gating scheme and Treg suppression assay**

**a)** Illustration of the gating scheme for splenic CD4^+^CD25^+^FoxP3^+^ Tregs. **b-d)** Illustrations of the Treg suppression assay: **(b)** isolation procedure and *ex vivo* carboxyfluorescein diacetate succinimidyl ester (CFSE) staining of total splenocytes from naïve mice (responder cells), **(c)** magnetic-associated cell sorting (MACS) separation of CD4^+^CD25^+^ splenocytes from male and female experimental mice of Experiment 1 including chronic subordinate colony housing (CSC) vs. single-housed control (SHC) male mice and social instability paradigm (SIP) vs. group-housed control (GHC) female mice, **(d)** *ex vivo* culturing of CFSE-stained “responder” splenocytes under basal (orange frame) and stimulated (anti-CD3 + CD28 (aCD3/28), grey frame) conditions in the absence or presence of CD4^+^CD25^+^ splenocytes from experimental male and female animals (green frame). Illustrations **(a-d)** were created with Biorender.com. For details see supplementary material and methods section 1.13 and 1.16.

**Fig. S3 | Intergenerational effects of repeated i.g. administrations of *Mycobacterium vaccae* ATCC 15483^T^ to female nulliparous C57BL/6N mice prior to mating on the negative consequences of chronic psychosocial stress during adulthood in male and female offspring (Experiment 1).**

**a-u)** show data from experimental male **(a-c)** and female **(d-u)** offspring of female nulliparous C57BL/6N mice that received administration of either borate-buffered saline (BBS)- or *M. vaccae* ATCC 15483^T^ (MvacATCC) after the exposure to chronic psychosocial stress (males: chronic subordinate colony housing (CSC) paradigm, females: social instability paradigm (SIP)). **a, o)** *Ex vivo* proliferation of CFSE-stained responder splenocytes from naïve male **(a)** or female **(o)** mice after exposure to 48 h basal and anti(a)CD3/28-stimulated conditions. Cells were not co-cultured with CD4^+^CD25^+^ T cells from experimental male or female mice, respectively. **b, p)** Proliferation of CFSE-stained responder splenocytes from naïve male or female mice (in % of aCD3/28-stimulated CFSE^+^ cells set to 100 %) co-cultured with CD4^+^CD25^+^ T cells from experimental male **(b)** or female **(p)** mice, respectively. **c, n)** Splenic CD4^+^CD25^+^FoxP3^+^ Tregs in percent (%) of CD4^+^CD25^+^ cells in male **(c)** or female **(n)** offspring. Relative **d)** adrenal, **e)** spleen, and **f)** thymus weight. **g)** Tibia length. **h)** Toll-like receptor (TLR)2, and **i)** TLR4. **j)** *Ex vivo* cell viability under basal and lipopolysaccharide (LPS) conditions at 0 µM corticosterone (CORT). **k)** Delta (LPS minus basal) *ex vivo* cell viability at 0 µM CORT. **l)** Delta *ex vivo* cell viability at 0.1 µM and 5 µM CORT in percent (%) of 0 µM CORT set to 100 %. **m)** Splenic CD4^+^CD25^+^FoxP3^+^ Tregs in percent (%) of all splenocytes. **q)** Treg suppressive capacity per female experimental mouse. **r)** Chemotactic activity of isolated neutrophils (*N* = 6 technical replicates per group). Percentage of splenic **s)** CD11b^+^Ly6G^+^Ly6C^–^ neutrophils, **t)** CD11b^+^Ly6G^+^Ly6C^+^ polymorphonuclear (PMN)-myeloid-derived suppressor cells (MDSCs), and **u)** CD11b^+^Ly6G^–^Ly6C^+^ monocytes/ monocyte-like (MO)-MDSCs. Data are presented as mean + SEM including individual values (dots). Blue bars: males, red bars: females. Non-hatched bars: BBS, hatched bars: MvacATCC; * *P* ≤ 0.05, ** *P* ≤ 0.01, *** *P* ≤ 0.001 vs. respective control condition (males: single-housed control (SHC), females: group-housed control (GHC)). *N* = 8 – 12 per group (Tab. S1). In case individual samples had to be excluded due to methodological reasons, this is reported in the supplementary materials and methods (Sections 1.15). Exact *P* values and statistical tests employed are provided in Tab. S2.

**Fig. S4 | Intergenerational effects of repeated i.g. administration of *Mycobacterium vaccae* ATCC 15483^T^ to adult nulliparous female C57BL/6N mice prior to mating on the community composition of the fecal microbiome prior to and after chronic psychosocial stress exposure during adulthood in male offspring (Experiment 2).**

**a, c)** Relative abundance of bacterial taxa (that were above the established cut-off for microbial abundance analyses (0.1%) in the majority of samples) found to be significantly different between the borate-buffered saline (BBS) and *M. vaccae* ATCC 15483^T^ (MvacATCC)-treated groups (orange bars: decreased in MvacATCC vs. BBS; green bars: increased in MvacATCC vs. BBS) prior to **(a)** or after **(c)** chronic psychosocial stress exposure. Taxa are sorted by phylum, order, family, genus and species. **b, d)** Correlation analysis of FoxP3^+^ Tregs and Treg suppression capacity per animal with orders (o_), and genera (g_) known to be short-chain fatty acid (SCFA) producers prior to **(b)** or after **(d)** chronic psychosocial stress exposure. Exact *P* values and statistical tests employed are provided either in Tab. S2 or directly in Fig. S4b, d.

**Fig. S5 | Effects of repeated i.g. administrations of *Mycobacterium vaccae* ATCC 15483^T^ to female nulliparous C57BL/6N mice prior to mating on maternal behavior (Experiment 1), composition of the fecal microbiome (Experiment 1) and the bodyweight of the offspring (Experiment 1).**

**a)** Number of pups per litter at birth. **b)** Body weight per litter at birth. **c)** Percentage (%) of observation intervals (daily observations between lactation day (LD) 1 to LD 6; 72 intervals per day; i.e. 4 intervals per 10 min for 3h) spent on nest, with total observation intervals set to 100 % (*N* = 8 litters per group). **d)** Latency to retrieve first pup in the pup retrieval test (PRT). **e)** Latency to retrieve pups back into the nest in the PRT. **f)** Body weight per litter at weaning. **g)** Numbers of pups per sex (males: blue bars, females: red bars). Data are presented as mean + SEM including individual values (dots). Non-hatched bars: borate-buffered saline (BBS), hatched bars: *M. vaccae* ATCC 15483^T^ (MvacATCC); ** *P* ≤ 0.01, ****P* ≤ 0.001 versus respective LD 1. *N* = 7 – 10 litters per group (Tab. S1). **h)** Relative abundance of bacterial taxa (that were above the established cut-off for microbial abundance analyses (0.1%) in the majority of samples) found to be significantly different between the borate-buffered saline (BBS) and *M. vaccae* ATCC 15483^T^ (MvacATCC)-treated groups. Taxa are sorted by family (f_) or genus (g_). In case individuals had to be excluded due to methodological reasons, this is reported in the supplementary materials and methods (Sections 1.2, 1.3). Exact *P* values and statistical tests employed are provided in Tab. S2.

**Fig. S6 | Effects of repeated i.g. administrations of *M. vaccae* ATCC 15483^T^ to female nulliparous C57BL/6N mice prior to mating on complement C3 expression in mammary glands of dams (Experiment 2).**

**a)** Experimental timeline of female C57BL/6N mice (Experiment 3). Female C57BL/6N mice received 3 weekly intragastric (i.g.; days –21, –14, –7) administrations of *M. vaccae* ATCC 15483^T^ (MvacATCC) or borate-buffered saline (BBS) prior to mating with a breeding male conspecific from days 0-3. All pregnant females received intraperitoneal (i.p.) injections with bromodeoxyuridine (BrdU) at day 4. After being single-housed on day 17 and giving birth to their offspring between days 19 and 23, dams and their offspring were euthanized between LDs 1 and 3. **b)** Relative C3 expression in mammary glands. Data are presented as mean + SEM including individual values (dots). Non-hatched bars: BBS, hatched bars: MvacATCC. *N* = 6 – 9 per group (Tab. S1). Illustration **(a)** was created with Biorender.com. Exact *P* values and statistical tests employed are provided in Tab. S2.
